# Designing Macrovoid-Free Polysulfone (PSf) Ultrafiltration (UF) Membranes with Tetraoxaundecane (TOU) Solvent

**DOI:** 10.1021/acsapm.6c02976

**Published:** 2026-09-28

**Authors:** Jadwiga Poniatowska, Kitty Nijmeijer, Zandrie Borneman

**Affiliations:** Membrane Materials and Processes, Department of Chemical Engineering and Chemistry, Eindhoven University of Technology, P.O. Box 513, 5600 MB Eindhoven, The Netherlands

**Keywords:** ultrafiltration (UF) membrane, nonsolvent induced phase separation (NIPS), tetraoxaundecane (TOU), polysulfone (PSf), water treatment

## Abstract

Formation of macrovoids in the membrane structure is a common occurrence in the process of nonsolvent-induced phase separation (NIPS), yet it is often undesired due to low structural homogeneity, poor performance, and introduction of weak mechanical points. Here, we introduce an ether-based solvent, 2,5,7,10-tetraoxaundecane (TOU), for fabrication of macrovoid-free polysulfone (PSf) ultrafiltration (UF) membranes. We investigate how strong polymer–solvent affinity, high chain entanglement, and high viscosity influence the phase-inversion process and promote the formation of homogeneous membrane structures during NIPS. Membranes prepared from PSf/TOU dope solutions exhibited stable sponge-like morphology, without macrovoids, in contrast to PSf membranes prepared from the more common solvent *N*-methyl-2-pyrrolidone (NMP), which typically display large fingerlike macrovoids. For PSf/TOU systems, mixing ethanol and water in the coagulation bath led to fabrication of porous, permeable membranes while maintaining the macrovoid-free morphology. Membrane performance was further optimized by changing the polymer concentration and the addition of a pore-forming agent in the dope solution. The best-performing membranes showed pure water permeance between 149 and 204 L·m^–2^·h^–1^·bar^–1^ and a bovine serum albumin rejection around 90%. This work establishes TOU as a previously unexplored yet effective solvent for NIPS fabrication of PSf UF membranes, enabling precise control over membrane structure and effective suppression of macrovoid formation and ultimately broadening the design space for PSf membranes.

## Graphical abstract

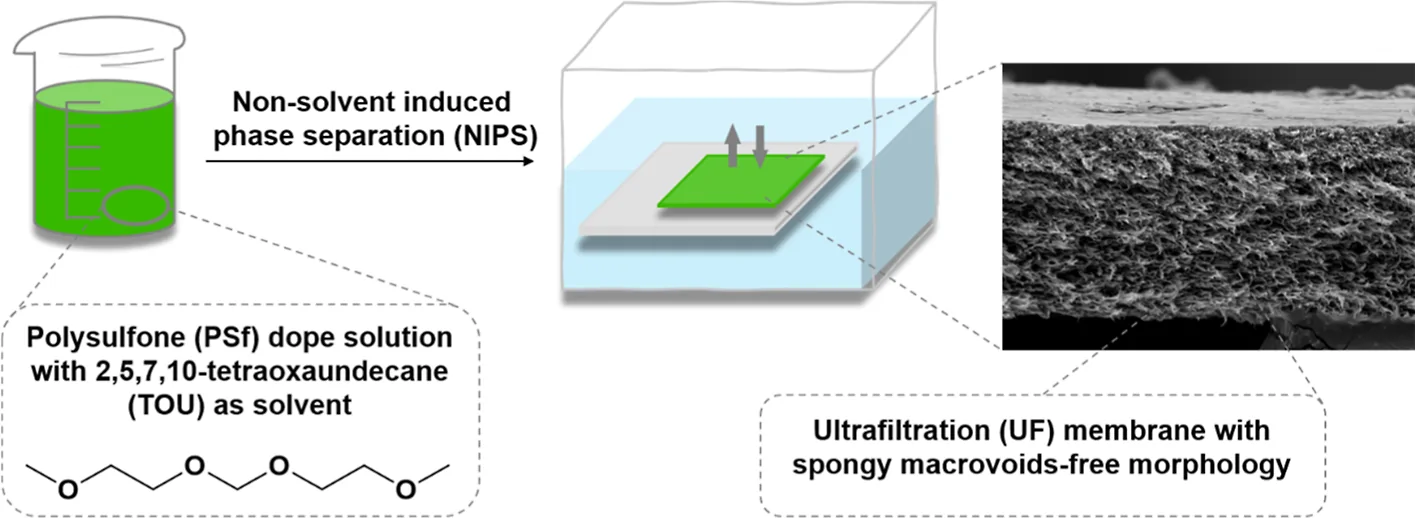

## 1 Introduction

Membranes are attractive solutions for designing continuous separation processes such as concentration of valuable components and their purification in industries including water treatment, food and beverages, petrochemicals, pharmaceuticals, catalysis, or metallurgy.^1^ Low energy usage, high efficiency, and the absence of phase changes are the factors contributing to the sustainability of membrane processes. Since 1962, when Sidney Loeb and Srinivasa Sourirajan invented nonsolvent-induced phase separation (NIPS),^2^ membranes prepared via this method have dominated the field. NIPS is a controlled method to induce polymer precipitation through the exchange of solvents and nonsolvents (often water). As the nonsolvent diffuses into the polymer solution, the polymer reaches its solubility limit and phase separates into a polymer-rich phase that forms the pore walls and a dilute polymer-poor phase that becomes the membrane pores.^3^

The final membrane morphology created during NIPS is strongly governed by the thermodynamical and kinetical aspects of the multicomponent system. When solvent–nonsolvent demixing occurs rapidly (e.g., due to good miscibility of solvent and nonsolvent or low dope solution viscosity), instantaneous demixing is favored, which frequently leads to asymmetric, porous, permeable structures with macrovoids, finger-like cavities, extending through the membrane flow section.^3^ Other reported conditions that contribute to macrovoid formation are shrinkage of the polymer matrix, surface tension gradient-induced convective flow, osmotic pressure differences, and concentration gradient-induced instability.^4^ While membranes with macrovoids are believed to have less resistance than symmetric macrovoid-free structures,^5^ they are generally undesired due to poor mechanical strength and compromised homogeneity and structural integrity.^6^ Consequently, controlling the phase inversion process to achieve more uniform, sponge-like, and macrovoid-free morphologies remains an important field in membrane science.

A key parameter affecting the phase separation behavior and, consequently, the formation of macrovoid-free membrane morphology is the solvent used in the dope solution. Solvent–nonsolvent interactions, solvent–polymer interactions, and dope solution viscosity all influence mass transfer during immersion-precipitation and thus the evolution of the membrane microstructure. As a result, the choice of solvent used in the dope solution plays a critical role in determining the phase inversion pathway and the resulting membrane morphology.

The solvents for NIPS must have both a high solvency power for the used polymers and be miscible with the nonsolvents used, typically, water. For this reason, the usage of a handful of polar aprotic solvents such as DMF, DMAc, or NMP is prominent in many large-scale industrial production sites. Zhou et al. showed that different solvents (namely, DMAc, NMP, DMF, and DMSO) have largely varying phase inversion behaviors for polysulfate (PSE) membranes, leading to production of dense or porous top layers, without altering other fabrication parameters.^7^ Moreover, apart from empirical studies, Hu et al. employed dissipative particle dynamics simulations to study the impact of nonsolvent–solvent affinity on membrane morphology and microstructure.^8^ These findings highlight that variations in solvent properties can significantly influence the resulting membrane structures.

Considering the key effect that the solvent has on membrane morphology, expanding the scope of suitable solvents beyond conventional DMF, DMAc, and NMP is of high importance for broadening of polymeric membrane spectrum. A promising solvent is 2,5,7,10-tetraoxaundecane (TOU), an ether-based polar aprotic solvent. Table 1 presents an overview of properties of TOU, in comparison with a more conventional solvent NMP, commonly used for membrane manufacturing. Notably, TOU is miscible with water, has a high boiling point and low vapor pressure favorable for safe processing, and is characterized by Hansen solubility parameters that suggest compatibility with a range of polymers, which makes it an attractive substitute for conventional solvents. However, TOU has recently been classified as suspected of damaging fertility and therefore is not a nontoxic or inherently greener alternative to conventional solvents. Nevertheless, TOU remains a valuable extension of the solvent portfolio available for NIPS membrane fabrication and provides an opportunity to investigate morphological control through the solvent properties. Very few mentions of TOU in academic literature have been reported but only for very different applications such as a diesel fuel oxygenate^9^ and as a solvent for the postcombustion CO_2_ capture process.^10^ TOU has been more prominently included in patents, e.g., as a component of paint removers,^11^ inks,^12^ primers,^13^ coatings,^14^ and as solvent for processing of polyurethanes.^15^ However, based on literature and patent searches, this is, to the best of our knowledge, the first publication that uses TOU as a solvent for membrane fabrication.

**Table 1:**
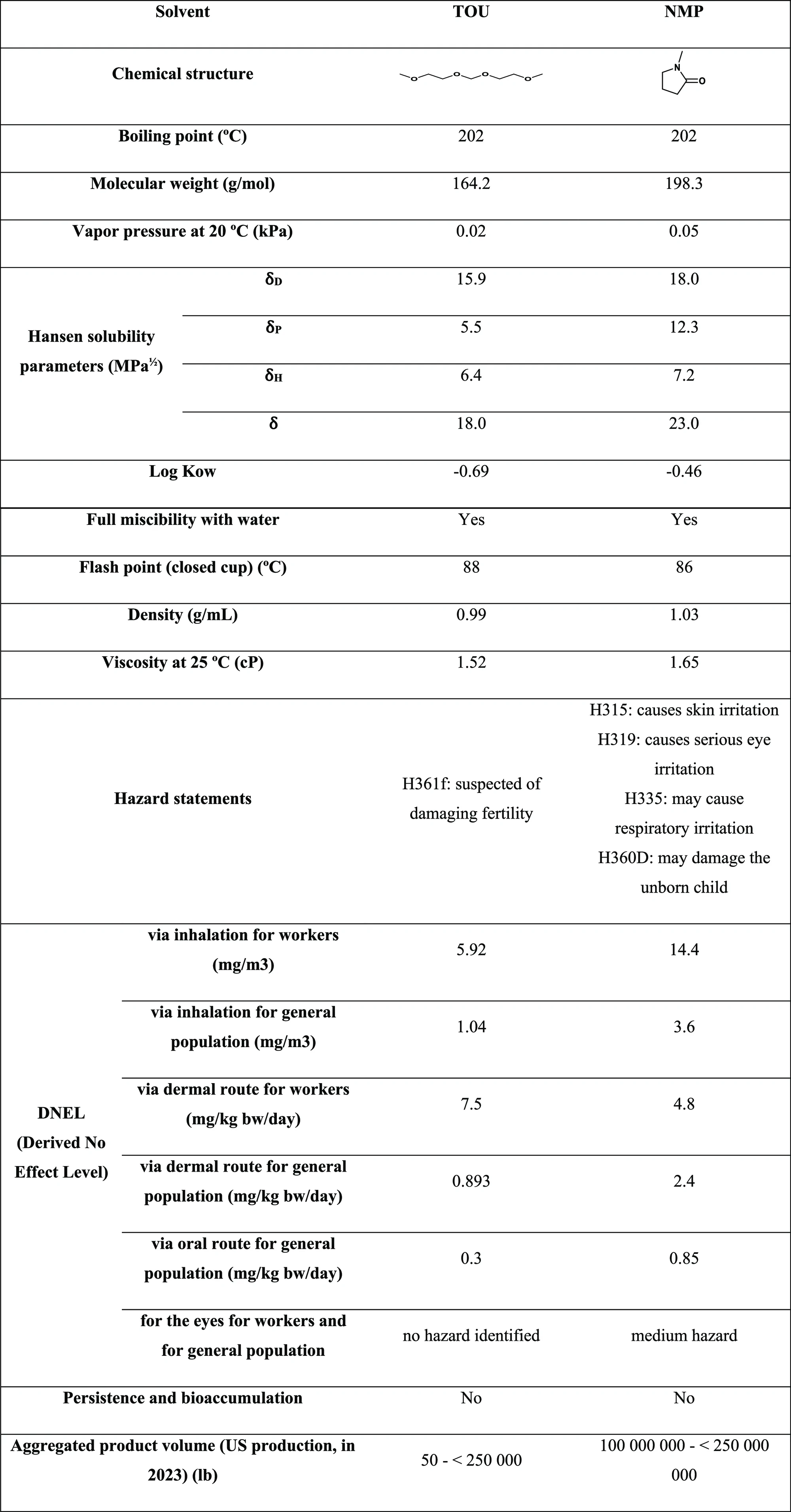
Overview of Properties of TOU and NMP^16–22^

This work demonstrates that TOU enables the fabrication of highly uniform, macrovoid-free sponge-like membranes. To elucidate this, particular attention is given to understanding how solvent properties influence the formation of membrane microstructures. Different processing parameters, namely, composition of coagulation bath, polymer concentration, and the addition of a pore-forming agent, are evaluated to explore the experimental operating space and to investigate both the window of opportunity and the versatility of TOU as a solvent for making controlled membrane morphologies. Ultrafiltration (UF) was selected as a proof-of-concept application to test the feasibility of TOU as it is a mature technology with industrial relevance.^23^ Polysulfone (PSf) is used as the membrane polymer due its superior mechanical, thermal, and chemical stability.^23^

## 2 Materials and Methods

### 2.1 Materials

Polysulfone (PSf, Udel P-3500 LCD MB7, molecular weight: 77–83 kg·mol^–1^)^24^ was kindly provided by Syensqo (Italy). Solvents for polymer dope solution fabrication, i.e., 2,5,7,10-tetraoxaundecane (TOU, which was kindly provided by Lambiotte & Cie, Belgium) and *N*-methylpyrrolidone (NMP, Biosolve, The Netherlands, 99.5%) were dried on molecular sieves prior to use. Polyethylene glycol with average Mn 600 (PEG600) as pore-forming agent was purchased from Sigma-Aldrich (Germany). Nonsolvents for NIPS were water (demineralized) and ethanol (VWR, France, 100.0%). Bovine serum albumin (BSA, heat shock fraction, pH 7, ≥98%) for rejection testing was purchased from Sigma-Aldrich (USA).

### 2.2 Cloud Point Measurements

PSf in TOU solutions were prepared at different concentrations (4, 8, 12, 16, 20 wt %). Nonsolvent (water or ethanol) was added dropwise into the PSf/TOU solutions under stirring, under ambient conditions (temperature ∼20 – 22 °C). The test was stopped when the solution remained visibly cloudy 24 h after the addition of nonsolvent. The compositions at which phase separation occurred were used directly to plot points of the binodal curve on the ternary phase diagram (PSf–TOU–nonsolvent). The measurements were performed only once at each concentration, hence no standard deviations were calculated.

### 2.3 Dope Solution Viscosity Measurements

Dynamic viscosity of all PSf dope solutions was measured using a rotational viscometer (Anton Paar ViscoQC 300), with 15 s^–1^ shear rate, at 20 °C, using a CC18 spindle. The reported viscosity values are an average of two independent measurements.

### 2.4 Thermodynamics

To understand the polymer solubility and the thermodynamics of NIPS with varying solvent/nonsolvent combinations, Hansen solubility parameters of specific compounds (Supporting Information 3) were taken into account and mutual affinity of two compounds (subscript 1 and 2, respectively) was calculated with the use of an interaction distance (*R*_*a*_) (eq 1)^25^

1 $$R_{a}=\sqrt{4{\left(\delta_{D1}-\delta_{D2}\right)}^{2}+{\left(\delta_{P1}-\delta_{P2}\right)}^{2}+{\left(\delta_{H1}-\delta_{H2}\right)}^{2}}$$

where δ_D_, δ_P_, and δ_H_ refer to dispersive interaction, polar interaction, and hydrogen bonding Hansen solubility parameter, respectively. The lower the interaction distance, the better chemical compatibility of two compounds.^25^

Relative energy differences (RED) for PSf and the solvents were calculated from the tabulated R_a_ values (eq 1) and the interaction radius of the PSf Hansen solubility sphere (*R*_0_ = 8).^26^ For RED <1, the solvent lies inside the Hansen solubility sphere of PSf and is predicted to be a good solvent.^26^

2 $$RED=\frac{R_{a}}{R_{0}}$$

### 2.5 Membrane Fabrication

PSf (and for some membranes PEG600) was added to a solvent (TOU or NMP) and dissolved for 3 days at room temperature on a roller bench. Prior to casting, the polymer solution was left stationary for minimum 1 day to degas. The polymer solution was cast with a 300 μm doctor blade onto a clean leveled A4-sized glass plate, under ambient laboratory conditions (temperature ∼20–22 °C, relative humidity ∼50%–70%). The polymer solution film was immersed in the coagulation bath (∼2 L, water/ethanol/water:ethanol mixture) immediately after casting, without a pre-evaporation step, to produce free-standing membranes via the NIPS process (Supporting Information 1). All membranes were kept in the coagulation bath for minimum 2 h and then stored in demineralized water prior to characterization and performance testing. The membrane codes (Supporting Information 2) used throughout the text are in the convention PSf concentration (15, 16, 17, or 18 wt %)—PEG600 concentration (0, 1, 2, 6 wt %)—solvent used (T refers to TOU, N refers to NMP)—coagulation bath composition (different vol % of W (water) or E (ethanol)). For example, a membrane denoted as 16-1-T-25W75E refers to a membrane formed from a dope solution containing 16 wt % PSf and 1 wt % of PEG600 dissolved in TOU and coagulated in a bath containing 25:75 v/v water/ethanol.

### 2.6 Membrane Characterization

The morphology of the membranes was analyzed by scanning electron microscopy (SEM; JEOL JSM-IT100) at an accelerating voltage of 10.0 kV after sputter-coating the samples with Pt at 40 mA for 120 s (JEOL JFC-2300HR).

The static contact angle of the membranes was tested using a Dataphysics OCA30 goniometer, using Milli-Q water as probe liquid (2 μL droplets), approximately 10 s after droplet administration to membrane surface. The reported contact angle was an average measurement of six droplets on different spots of the membrane. The errors represent the standard deviation of those six measurements.

Dynamic mechanical analysis (DMA), specifically stress–strain curves, were collected with a DMA Q800 (TA Instruments) at 50 °C, which is well below the glass transition temperature of PSf, yet moderately elevated, potentially relevant for industrial applications. The measurements were performed in air with a preload force of 0.01 N, on rectangular membrane samples, with length of ∼20 mm, width of 5.3 mm, and thickness of 0.05–0.13 mm. The samples were subjected to a force ramp of 3.0 N·min^–1^. The measurements were performed once for each membrane sample; therefore, standard deviations were not calculated.

Membrane thickness was determined using a digital micrometer (Helios Preisser), with a precision of 0.001 mm. The errors represent the standard deviation of thickness measured at 4 different spots on the membrane.

Membrane porosity was determined using a dry-wet gravimetric method.^27^ Circular membrane pieces with a diameter of 22 mm were dried at 40 °C under vacuum for 24 h and weighed. Then, they were immersed in water for 3 days, quickly blotted with a tissue to remove adhered water and immediately weighed. The porosity (%) was calculated with eq 3

3 $$porosity=\frac{\frac{w_{w}-w_{d}}{\rho_{w}}}{\frac{w_{w}-w_{d}}{\rho_{w}}+\frac{w_{d}}{\rho_{p}}}\cdot 100\%$$

where *w*_*w*_ is the wet weight (g), *w*_*d*_ is the dry weight (g), ρ_*w*_ is the density of water (0.997 g/mL), and ρ_*p*_ is the density of PSf (1.240 g/mL).^28^ The errors represent the standard deviation from 3 separate membrane pieces.

### 2.7 Membrane Filtration Performance

The membrane performance was tested at room temperature primarily using a high-throughput dead-end filtration stirred SPIDER cell (Porometer, Belgium) (SI 4). The cell allows for simultaneous measurement of 16 membrane samples with one single feed (600 mL) but separate permeate outlets. The active membrane filtration area was 2.0×10^–4^ m^2^. The applied pressure was 2 bar. To avoid initial nonequilibrium conditions, collection of permeate was only started after 10 min of permeation (i.e., data collection was commenced only after 10 min of permeate flux stabilization), after which it was performed continually for 1 h at steady-state (Supporting Information 5). Pure water permeance (L·m^–2^·h^–1^·bar^–1^) was determined using eq 4

4 $$permeance=\frac{V}{A\cdot t\cdot P}$$

where *V* is the volume of the collected permeate (L), *A* is the active membrane filtration area for one single membrane (*m* ^2^), *t* is the time of the permeance measurement (*h*), and *P* is the applied transmembrane pressure (bar).

The BSA rejection of the membranes was determined using a BSA (0.5 g/L) aqueous feed solution (prepared with demineralized water). BSA is a protein with molecular weight of 67 kDa, dimensions of an individual molecule of 14 nm × 4 nm × 4 nm, and overall folded size in the range of 5–10 nm at pH ∼4–8 in N form,^29–31^ therefore falling in the lower size range of UF membranes (2–100 nm).^23^ The BSA rejection (%) was calculated using eq 5

5 $$rejection=\frac{C_{f}-C_{p}}{C_{f}}\cdot 100\%$$

where *C*_f_ and *C*_p_ are the BSA concentrations (g/L) in the feed and permeate, respectively. For membranes with very low permeances, BSA rejection was not measured due to insufficient permeate volume. The number of membranes tested at once was adjusted to prevent high (more than 200 mL) feed volume reduction, and hence significant changes in *C*_f_. The concentrations were measured using UV–vis spectrophotometry (UV-1280, Shimadzu), at 279 nm, with the baseline of pure water, using an absorbance vs concentration calibration curve (Supporting Information 6). The reported permeances and rejections were an average of three separate membrane pieces, with error bars corresponding to the standard deviation.

To mimic more realistic industrial conditions, a cross-flow filtration was carried out with a best-performing membrane. An MMS triple system (MMS AG Membrane Systems, Switzerland) with a feed volume of 900 mL allowed for simultaneous testing of three larger membrane samples (2.8×10^–3^ m^2^) at the same 2 bar transmembrane pressure as with dead-end filtration. The cross-flow velocity was 0.61 m·s^–1^, and the circulation flow rate was 63 L·h^–1^. The order of measurements followed 10 min pure water, 15 min BSA solution, 30 min pure water, 30 min pure water, 30 min pure water, and 30 min BSA solution. In between the measurements, the cell was depressurized, emptied from the remaining solution, and refilled with a fresh solution. The pure water permeance as well as the BSA rejection was calculated according to eqs 4 and 5 above.

## 3 Results and Discussion

### 3.1 Optimizing PSf/TOU Membranes by Adjusting the Water–Ethanol Ratio in the Coagulation Bath

Typically, UF membranes prepared via NIPS are coagulated in water due to its environmental compatibility, although other nonsolvent types have been explored in literature.^23^ Here, the morphology, performance, and properties (Figure 1, Table 2) of membranes fabricated from PSf/TOU dope solutions and coagulated in water, ethanol, or mixtures of water and ethanol at different ratios were evaluated and correlated to Hansen solubility parameters for different components in order to investigate the most compatible nonsolvent type.

**Figure 1:**
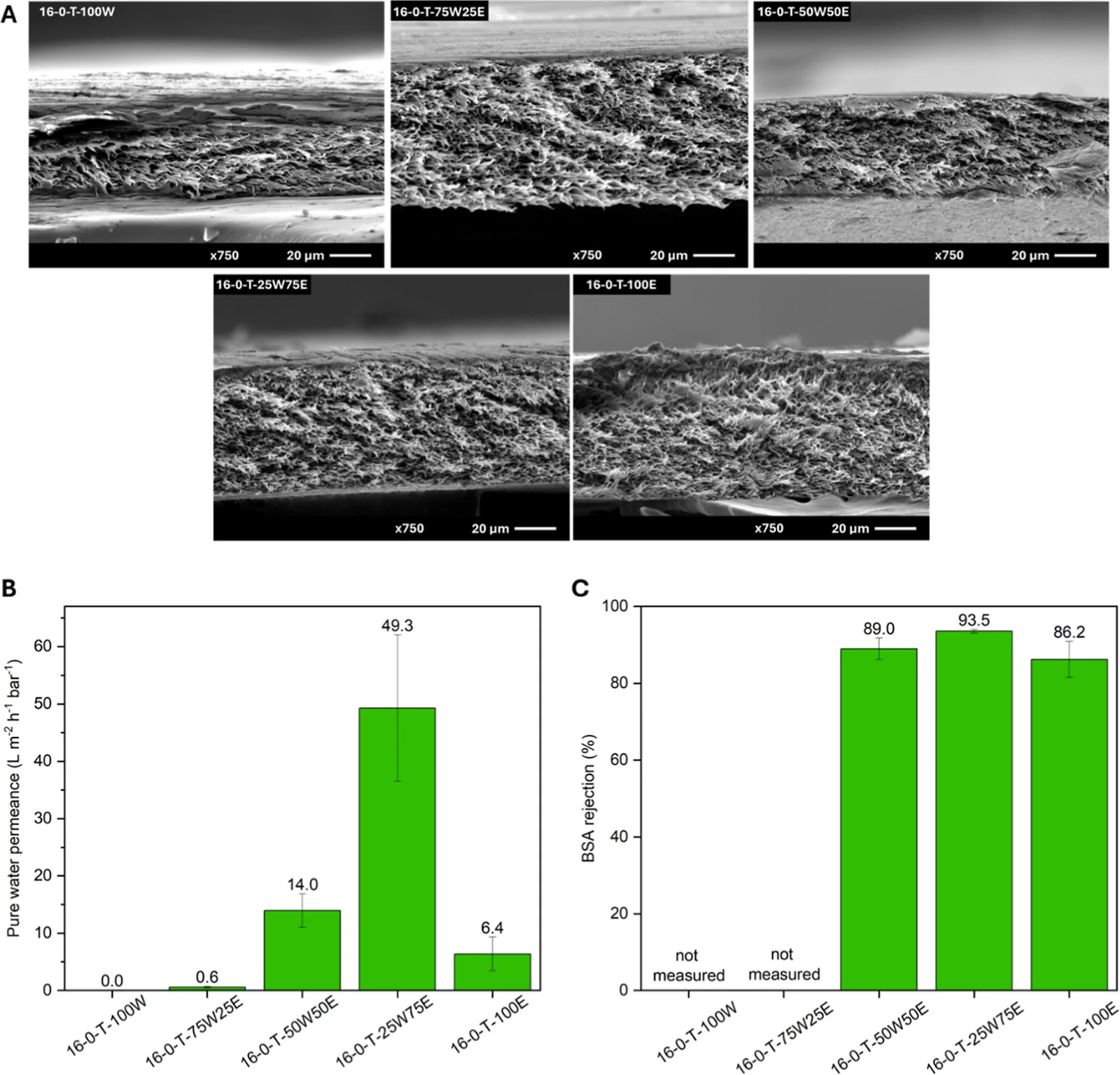
(A) Morphology of the PSf/TOU membranes with varying water/ethanol (100:0, 75:25, 50:50, 25:75, 0:100) ratios in coagulation bath. SEM images, full cross sections, magnification ×750. (B) Pure water permeance and (C) BSA rejection of PSf/TOU membranes with varying water/ethanol ratios in the coagulation bath. When permeance was too low for sufficiently accurate measurement, BSA rejection was reported as “not measured”.

**Table 2:**
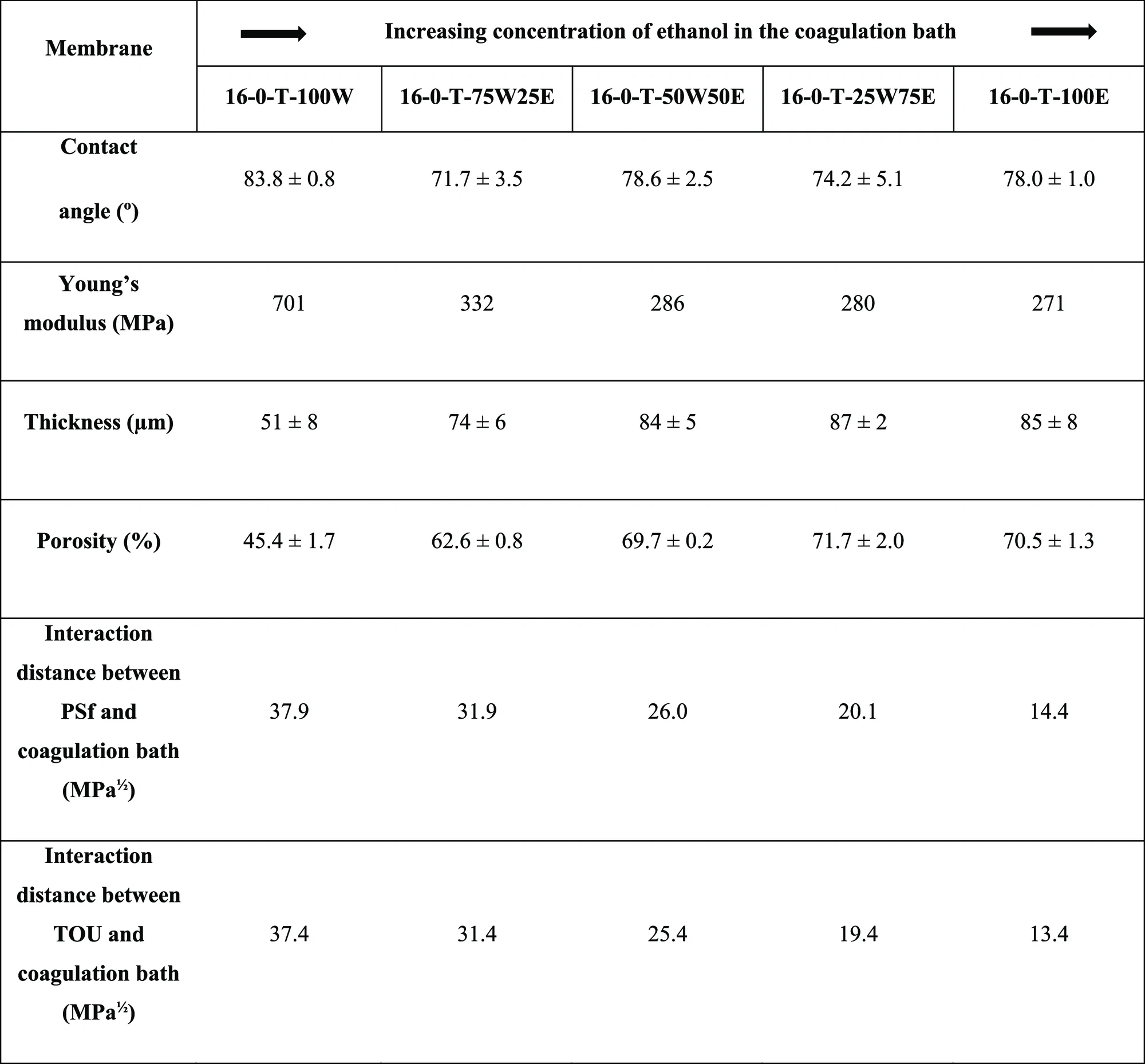
Properties (Contact Angle, Young’s Modulus, Thickness, Porosity) of PSf/TOU Membranes with Varying Water/Ethanol Ratios in the Coagulation Bath. Interaction Distances Based on Hansen Solubility Parameters for PSf and TOU and Varying Nonsolvents

SEM cross-sectional images (Figure 1A) show that all tested membranes exhibit a morphology consisting of a denser skin layer and spongy substructure with a gradient in porosity, without the presence of macrovoids. For pressure-driven membrane processes, the presence of macrovoids is undesired since these are more prone to collapse and defect formation,^32^ making the presented PSf membranes manufactured from TOU highly suitable for filtrations at higher pressures when compared with PSf-NMP macrovoid-containing membranes. Interestingly, irrespective of the nonsolvent used, all of the membranes show a similar overall cross-sectional morphology. This suggests that the morphology is truly the effect of PSf-TOU interactions, rather than the effect of the nonsolvent. Moreover, the images show that TOU is a suitable versatile solvent for preparing sponge-like morphologies across different coagulation bath compositions.

In spite of similar macrovoid-free cross-sectional morphologies of the presented membranes, there are differences in top layer properties and resulting permeance and retention. The pure water permeance (Figure 1B) gradually increases upon increasing ethanol content in the coagulation bath, from an almost impermeable membrane coagulated in pure water (16-0-T-100W) to a well-permeable membrane coagulated in a 25:75 v/v water/ethanol mixture (16-0-T-25W75E). In addition, Figure 1C shows that the membranes 16-0-T-50W50E, 16–0-T-25W75E, and 16-0-T-100E all reject around 90% BSA. This strong evidence points to a selective layer with pore sizes that are either at or just under the size of a BSA molecule, confirming the absence of significant defects that would compromise selectivity.

The solvent–nonsolvent interactions define the thermodynamic driving force for phase separation, while the rate of solvent–nonsolvent exchange governs whether instantaneous or delayed demixing occurs.^3,32^ Generally, when the mutual affinity of the solvent with the nonsolvent is higher (i.e., TOU and water–ethanol mixtures), their solvent exchange fluxes increase, inducing more instantaneous demixing, a higher top-layer porosity, and more permeable membranes.^3^ TOU has higher affinity with ethanol than with water as the Ra between TOU and water is 37.4, whereas the Ra between TOU and ethanol is 13.4 (Table 2). The observed increasing trend in pure water permeance from the 16-0-T-100W to the 16-0-T-25W75E membrane can therefore be associated with the increased affinity of TOU to the coagulation bath. However, the membrane coagulated in pure ethanol (16-0-T-100E) exhibits a subsequent drop in pure water permeance, in spite of an even higher affinity between TOU and pure ethanol. Simultaneously, parallel to increased affinity between TOU and the water–ethanol mixture, also the affinity between PSf and the water–ethanol mixture increases with increasing ethanol fraction, i.e., ethanol is not an effective nonsolvent for PSf, which favors delayed demixing and leads to lower pure water permeance.^3^ A similar trend of PSf membranes coagulated in different ratios of water/ethanol was observed by Hackett et al.^23^ When PSf/γ-valerolactone dope solution films were coagulated either in pure water or pure ethanol, impermeable membranes formed, whereas using water/ethanol mixtures resulted in significant increases in flux.^23^

To not rely solely on Hansen solubility parameters, cloud point measurements were performed, and a zoom of the ternary phase diagram of PSf-TOU-nonsolvent (ethanol or water) was created (Figure 2). The empirical results confirm that water is a stronger nonsolvent than ethanol since for a 16 wt % PSf solution in TOU, less than 1.3 wt % of water and 2.9 wt % of ethanol were needed to induce phase separation in the system.

**Figure 2:**
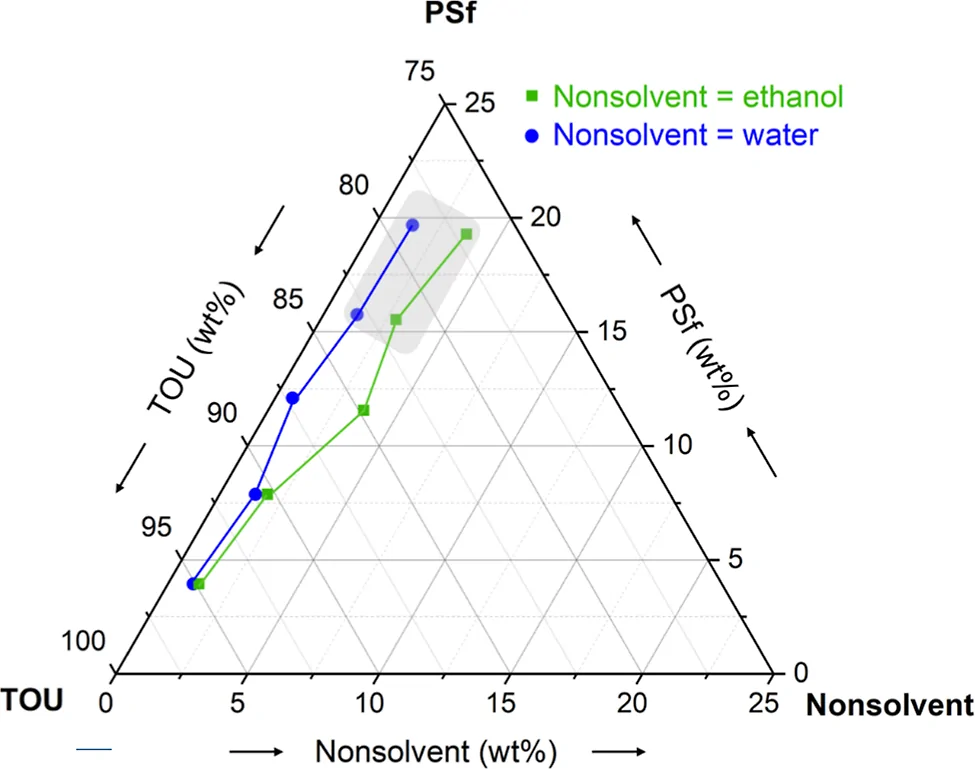
Zoom of the ternary phase diagram of PSf, TOU, and nonsolvent (water in blue, ethanol in green). The gray shaded area shows a relevant PSf concentration range (typically about 15%–20% polymer) for membrane fabrication, where water is a significantly stronger nonsolvent than ethanol.

The contact angle of all membranes (Table 2, Supporting Information 7) is in the range of 71°–84°, hence all membranes have a slight hydrophilic character due to the aryl-sulfonyl-aryl subunits of PSf. The measured contact angles are in line with the previously reported values: Xie et al. observed a PSf membrane contact angle of 80.7° ± 2.5°,^33^ whereas Prabhu et al. reported a contact angle of 70° ± 1°.^34^ The 16-0-T-100W membrane exhibits the highest contact angle among the tested samples, i.e., the least hydrophilic. All membranes are fabricated from the same polymer, without the use of additives, hence any differences in the observed contact angle are due to membrane surface morphology rather than surface chemistry. For rough and porous surfaces, the measured apparent contact angle can be affected by roughness-induced wetting phenomena, as described by the Wenzel model.^35^

The Young’s modulus (Table 2, Supporting Inforamtion 8) (or tensile modulus) is a measure of the resistance of the membranes to elastic deformation under load and was retrieved from the initial linear portion of the stress–strain graphs.^3^ Young’s moduli consistently decrease from 701 to 271 MPa upon increasing ethanol concentration in the coagulation bath. Gzara et al. fabricated polymeric membranes via NIPS (using NMP as solvent), which exhibited Young’s moduli between 75.6 and 139.6 MPa,^36^ whereas Peng et al. reported Young’s moduli in the range of 154.6 and 249.4 MPa for NIPS membranes (using DMSO as the solvent).^37^ In comparison to typical literature values, the higher Young’s modulus of 16-0-T-100W membrane is characteristic for stiff materials that tend to be more brittle and fail under lower loads. Hence, remaining PSf membranes prepared in coagulation baths consisting of water/ethanol mixtures have more balanced mechanical properties, possibly advantageous for handling and operation.

With respect to membrane thickness (Table 2), all polymer solutions were cast with a 300 μm doctor blade knife, which is within the typical casting range (e.g., Peng et al. used a 500 μm knife,^37^ Plisko et al. used a 250 μm knife,^38^ whereas Young et al. used a 150 μm knife).^39^ Also, all reported membranes, in this section, were made from dope solutions with 16 wt % PSf concentrations, hence any difference in the final dry thickness is a result of the phase separation process. The 16-0-T-100W membrane has a considerably lower thickness (51 μm) than the membranes coagulated in ethanol or water/ethanol mixtures. Since water and TOU have poor affinity (high R_a_), the presence of TOU can locally delay solvent–nonsolvent exchange, leading to delayed demixing in TOU-rich regions. As a result, membrane formation and solidification is slower, PSf has more time to rearrange, creating a denser structure and a thinner membrane.

A similar trend was observed with regards to membrane porosity (Table 2). The 16-0-T-100W membrane, due to delayed demixing, exhibits the lowest overall volume porosity (45.4%), whereas addition of ethanol to the coagulation bath leads to production of membranes with increasingly higher porosity. The increase in overall porosity indicates a more open membrane substructure; however, because the measured porosity represents the entire membrane volume, it cannot by itself predict the resistance of the selective top layer or the resulting pure water permeance. Moreover, the membrane porosity can also be correlated with the above-mentioned mechanical properties (Table 2) since typically, high-porosity materials exhibit low Young’s moduli.^40^

Overall, by changing the composition of the coagulation bath with different volume fractions of water and ethanol, various PSf/TOU membranes can be fabricated in a facile manner with a range of properties. The comparison between the 16-0-T-100W and 16-0-T-25W75E membranes shows that by replacing pure water with a 25:75 water/ethanol mixture in the coagulation bath makes it possible to change from a membrane that is almost impermeable, brittle, and with a low porosity to a permeable and more flexible effective UF membrane, with more favorable water permeance and BSA rejection, while still retaining a macrovoid-free morphology.

### 3.2 Comparison between Membranes Fabricated from TOU and NMP

As all PSf membranes prepared with TOU exhibited spongy macrovoid-free microstructures, in spite of varying solvent–nonsolvent interactions, further differences were investigated with PSf membranes prepared with the conventional solvent, NMP (Figure 3 and Table 3), to study the causes of macrovoid suppression.

**Figure 3:**
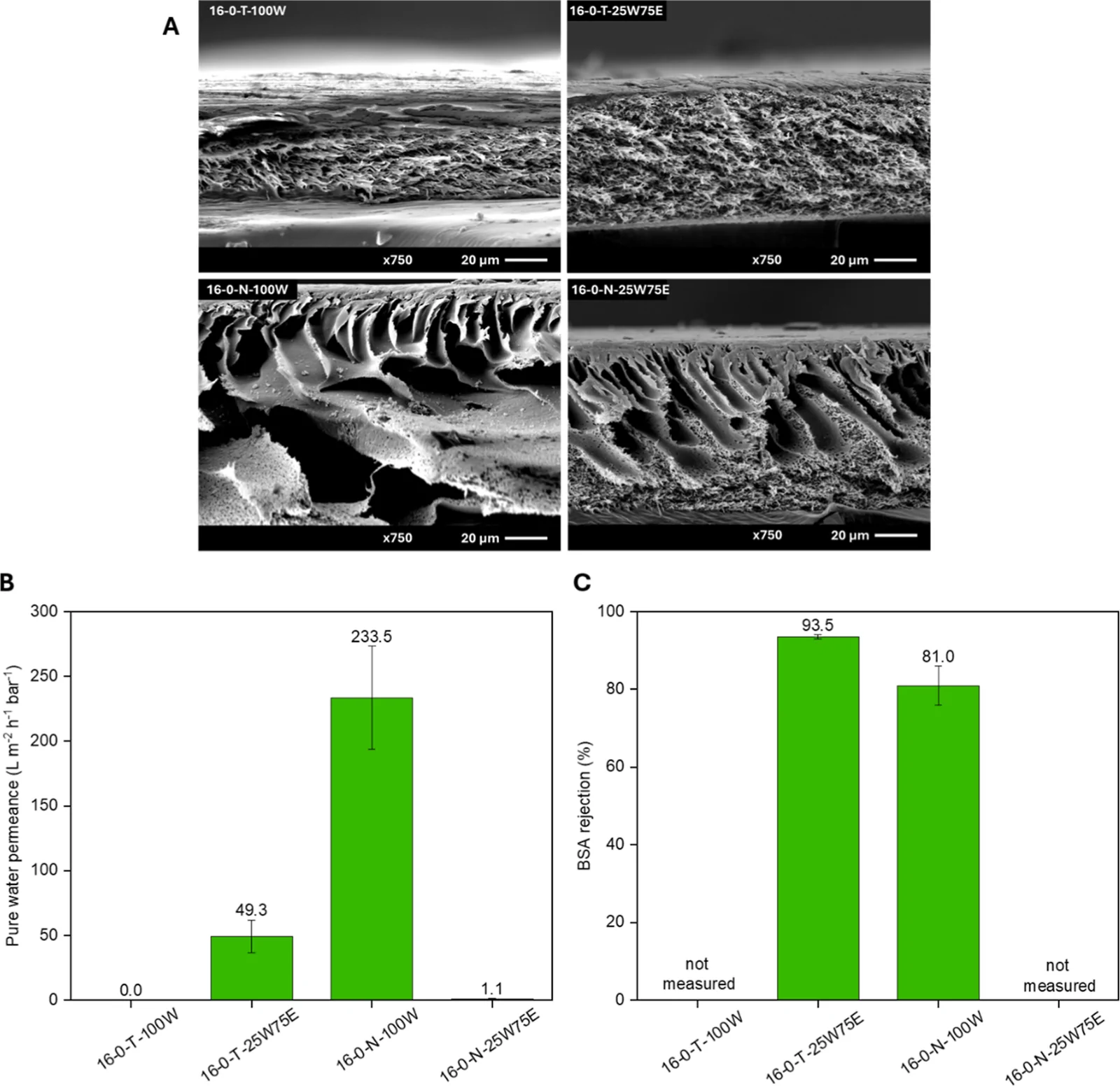
(A) Morphology of the PSf/TOU and PSf/NMP membranes coagulated either in pure water or 25:75 water/ethanol bath. SEM images, full cross sections, magnification ×750. (B) Pure water permeance and (C) BSA rejection of PSf/TOU and PSf/NMP membranes coagulated either in pure water or 25:75 water/ethanol bath. When permeance was too low for sufficiently accurate measurement, BSA rejection was reported as “not measured”.

**Table 3:** Properties (Viscosity, Contact Angle, Young’s Modulus, Thickness, Porosity) of PSf/TOU as well as PSf/NMP Dope Solutions and Membranes, Coagulated Either in Pure Water or 25:75 Water/Ethanol Bath. Interaction Distances Based on Hansen Solubility Parameters for PSf, TOU, NMP, and Varying Nonsolvents (Water or 25:75 Water/Ethanol)

	TOU		NMP	
membrane	16-0-T-100W	16-0-T-25W75E	16-0-N-100W	16-0-N-25W75E
dope solution dynamic viscosity at 20 °C (mPa·s)	1136 ± 4	1136 ± 4	741 ± 69	741 ± 69
contact angle (°)	83.8 ± 0.8	74.2 ± 5.1	68.5 ± 3.5	74.5 ± 3.9
Young’s modulus (MPa)	701	280	145	218
thickness (μm)	51 ± 8	87 ± 2	126 ± 3	86 ± 3
porosity (%)	45.4 ± 1.7	71.7 ± 2.0	79.0 ± 0.2	73.5 ± 0.7
interaction distance between PSf and coagulation bath (MPa^1/2^)	37.7	19.9	37.7	19.9
interaction distance between solvent and coagulation bath (MPa^1/2^)	37.4	19.4	35.6	18.6
interaction distance between PSf and solvent (MPa^1/2^)	4.8	4.8	8.0	8.0

The most striking difference between PSf membranes fabricated with TOU and NMP is that the latter has large amounts of macrovoids, whereas the former exhibits spongy substructures (Figure 3A). The observed morphology is present irrespective of the nonsolvent used in the coagulation bath, suggesting that it is truly the effect of the solvent (TOU or NMP). PSf membranes fabricated with NMP also have higher overall porosity (Table 3) irrespective of the nonsolvent used, than PSf membranes fabricated with TOU, which can also be attributed to the presence of macrovoids—hollow voids/pores in the macro scale in the membrane structure.

Based on thermodynamic considerations derived from Hansen solubility parameters, TOU is a better solvent for PSf (R_a_ PSf-TOU = 4.8, RED = 0.6) than NMP (R_a_ PSf-NMP = 8.0, RED = 1) (Table 3). A better solvent such as TOU leads to better interactions with the polymer in the dope solution, more expanded polymer chains with higher hydrodynamic volumes, which may result in increased interchain contacts and entanglements.^41,42^ The better interactions between PSf and TOU than PSf and NMP are also confirmed with dope solution viscosity: 16 wt % PSf solution in TOU has a significantly higher viscosity than in NMP (1136 vs 741 mPa·s (Table 3)), in spite of pure NMP having slightly higher viscosity than TOU (1.65 vs 1.52 mPa·s (Table 1)). In polymer solutions, stronger interactions, differences in polymer conformation, interchain interactions, and more chain entanglements are known to significantly increase viscosity.^43^ High solution viscosity and high degree of chain entanglement can contribute to suppression of the occurrence of macrovoids,^44,45^ hence explaining the stark difference in morphologies between membranes prepared from PSf/TOU (more thermodynamically controlled) or PSf/NMP (more kinetically controlled) dopes, irrespective of nonsolvents used. To further assess whether the absence of macrovoids in the PSf/TOU membranes can be explained solely by high dope solution viscosity, an additional 14-0-25W75E membrane was prepared from a 14 wt % PSf/TOU dope solution. Although the viscosity of the 14 wt % PSf/TOU dope (562 mPa·s) was lower than that of the 16 wt % PSf/NMP dope (741 mPa·s), the resulting membrane still exhibited a homogeneous, spongy, and macrovoid-free microstructure (Supporting Information 9). This demonstrates that high bulk dope viscosity alone is not necessary for macrovoid suppression in the TOU-based system, and likely results from an interplay of polymer–solvent interactions and entanglements. Cloud point measurements and corresponding ternary phase diagrams provide an additional indication that the solvent highly influences the phase separation pathway, as for the PSf/NMP system, 5–10 wt % water is required to induce phase separation, as shown by Lau et al.,^46^ whereas for the PSf/TOU system, only 0.7–1.4 wt % water is needed (Figure 2). To directly establish the entanglement state of PSf chains in TOU and NMP, further work is recommended to include rheological frequency sweeps to fully resolve storage modulus, loss modulus, and relaxation time. Similarly, to fully understand the differences in fabrication process for PSf/TOU and PSf/NMP membranes, phase separation kinetics (e.g., through light transmission measurements) are a useful addition, which were out of scope in the present study.

The trend in pure water permeance (Figure 3B) between membranes fabricated from NMP and TOU shows an interesting inverse trend: the 16-0-T-100W membrane is less permeable than the 16-0–25W75E membrane, whereas the 16-0-N-100W membrane is more permeable than the 16-0-N-25W75E membrane. As previously discussed in Section 3.1., higher permeances originate from faster demixing, and a balance between good miscibility of solvent and nonsolvent, as well as poor miscibility between polymer and nonsolvent is required. Ethanol is better miscible with typical polar aprotic solvents than water (faster phase inversion), but also better miscible with PSf than water (slower phase inversion). Hence, depending on the exact polymer–solvent–nonsolvent system, different proportions of water/ethanol lead to different structures and thus permeances. For the PSf/TOU/nonsolvent system, the higher relative proportion of ethanol/water in the coagulation bath leads to production of membranes with higher permeability, but for the PSf/NMP/nonsolvent system, pure water in the coagulation bath produces higher permeability membranes. Hence, Hansen solubility parameters cannot be used alone for defining and/or predicting morphology, properties and performance of the PSf membranes. A similar conclusion was raised by Hackett et al. for PSf membranes fabricated with cyrene and γ-valerolactone: that a balance should be struck between high miscibility of ethanol with a solvent, and the high nonsolvent strength of water, as well as that different ethanol/water compositions should be explored to find the optimal balance for the desired performance.^23^

Overall, it is shown that the suppression of macrovoids in the membranes prepared from TOU stems from better PSf solvency power than NMP, which serves as an important parameter beyond simple solvent–nonsolvent interactions for guiding phase inversion.

### 3.3 Effect of PSf Concentration

An important and versatile parameter for changing the membrane morphology and performance is the polymer concentration. Membranes with varying PSf content in the TOU dope solution were fabricated and characterized (Figure 4, Table 4).

**Figure 4:**
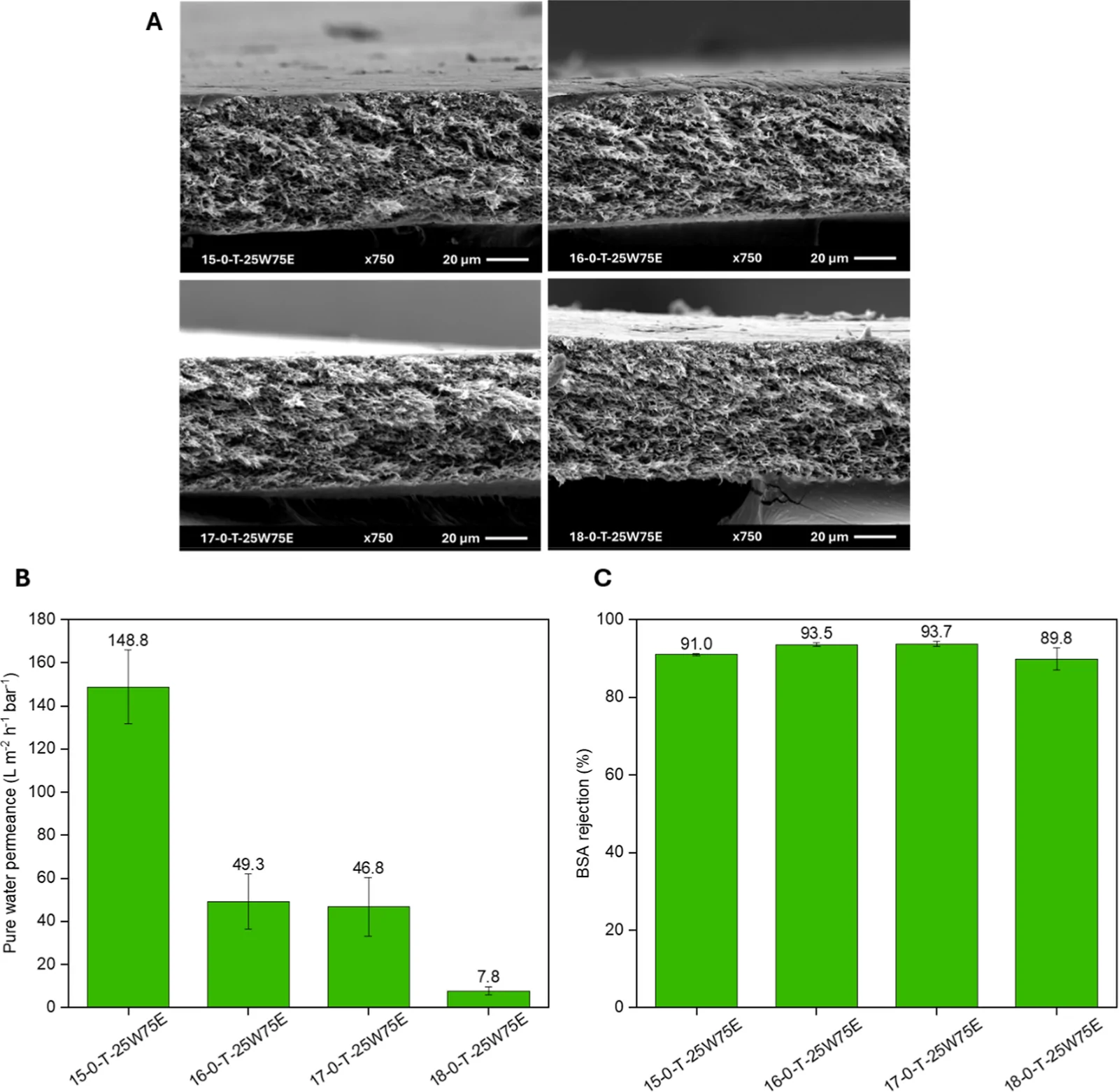
(A) Morphology of the PSf/TOU membranes prepared from dope solutions with varying polymer concentration (15, 16, 17, 18 wt·%). SEM images, full cross sections, magnification ×750. (B) Pure water permeance and (C) BSA rejection of PSf/TOU membranes with varying polymer concentration.

**Table 4:**
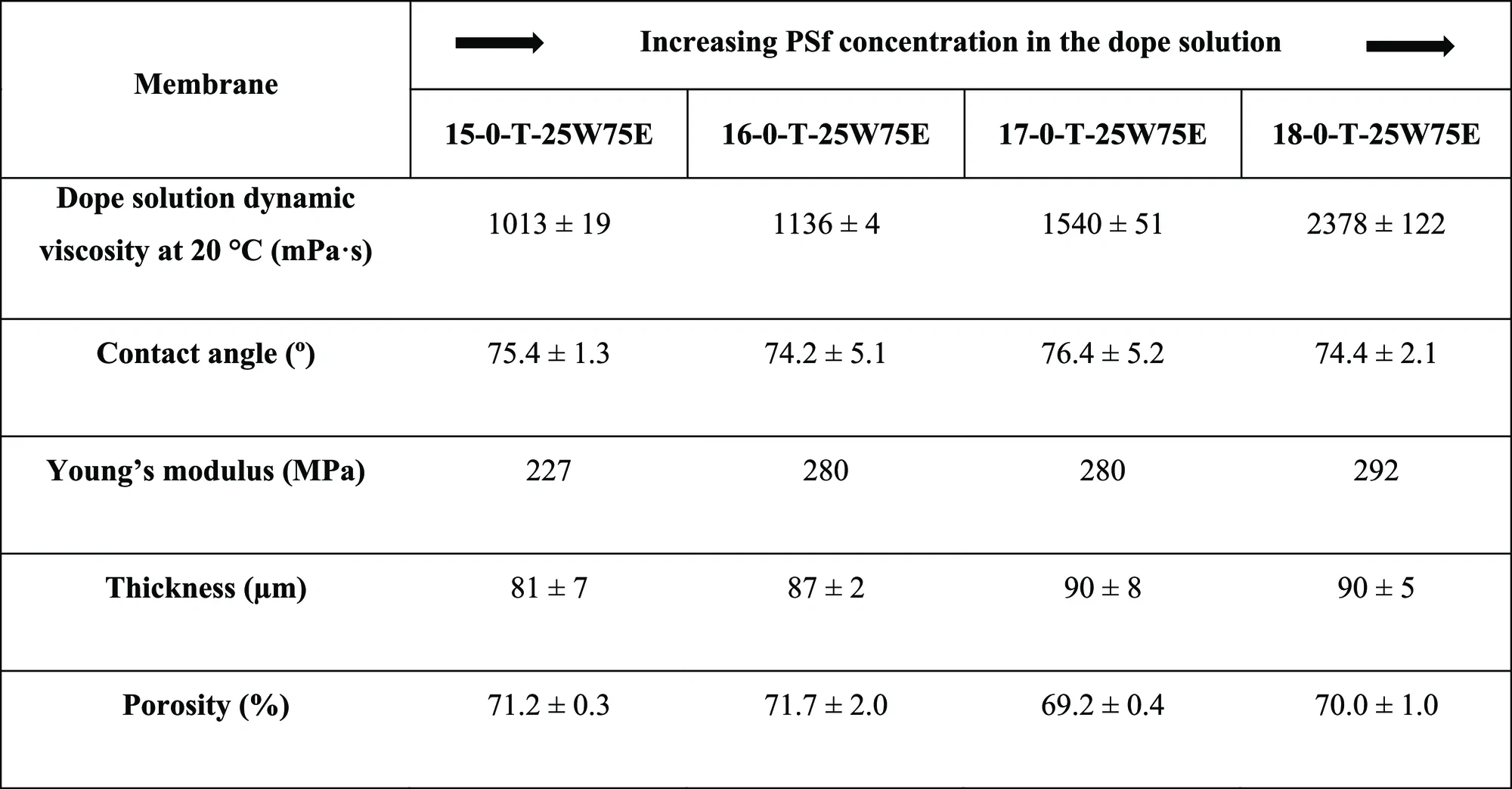
Properties (Viscosity, Contact Angle, Young’s Modulus, Thickness, Porosity) of PSf/TOU Dope Solution and Membranes with Varying Polymer Concentrations

The morphology of all membranes with varying PSf concentrations (Figure 4A) is similar, with spongy substructures and denser top layers, although the 18-0-T-25W75E membrane is visibly more uniform and has fewer random voids and fewer larger pores than the 15-0-T-25W75E membrane.

Membranes with lower PSf concentration in the dope solution exhibit a considerably higher pure water permeance than membranes with higher PSf concentration (Figure 4B). The trend in pure water permeance is in line with what is typically observed in the NIPS process: lower polymer concentration and lower viscosity of the original dope solution lead to a lower polymer concentration at the interface, lower mass flow resistance for solvent–nonsolvent exchange, faster kinetics of phase separation, and as a result, higher porosity of the top layer.^3,47^ Relatively small changes in the PSf concentration in the dope solution (from 18 to 15 wt %) show large improvements in performance, approximately a 19-fold increase in pure water permeance. Thus, decreasing the PSf concentration in the dope solution is a facile manner of significantly improving permeance.

The increase in pure water permeance with decreasing PSf concentration in the dope solution also does not negatively impact BSA rejection results (Figure 4C), suggesting that mostly the smallest pores of the top layer are affected, without an increase above the size of the model solute. Hence, within the UF operational window, the 15-0-T-25W75E membrane shows superior performance.

As the performance of the 15-0-T-25W75E membrane was the most favorable, it was also studied in a cross-flow configuration. Cross-flow, also known as tangential flow filtration, prevents the infinite growth of a fouling layer on the top of the membrane surface.^48^ The initial pure water permeance (first 10 min in Figure 5) of the 15-0-T-25W75E membrane is on average similar to the permeance observed with the dead-end filtration cell (149 L·m^–2^·h^–1^·bar^–1^, A), in spite of larger error bars possibly correlated with the hydrodynamics in the cross-flow setup being more sensitive to drift. The BSA solution exhibits much lower permeance (next 15 min in Figure 5) than pure water at the same transmembrane pressure, possibly due to fouling and higher osmotic pressure. Three rounds of in situ water cleaning (3 × 30 min in Figure 5) do not however increase the permeance back, suggesting that fouling occurs, irrespective of operating in the cross-flow regime. The second round of BSA solution permeation (last 30 min in Figure 5) led to a similar permeance, showing that no further decrease in performance occurs at prolonged operation, and an even slightly higher rejection profile is observed. For the 15-0-T-25W75E membrane to be more effective, additional cleaning methods should be considered for the future, such as backflushing,^49^ use of alkaline (NaOH) or oxidizing (NaClO) reagents,^50^ or further chemical modifications, increasing surface hydrophilicity and decreasing fouling.^49^

**Figure 5:**
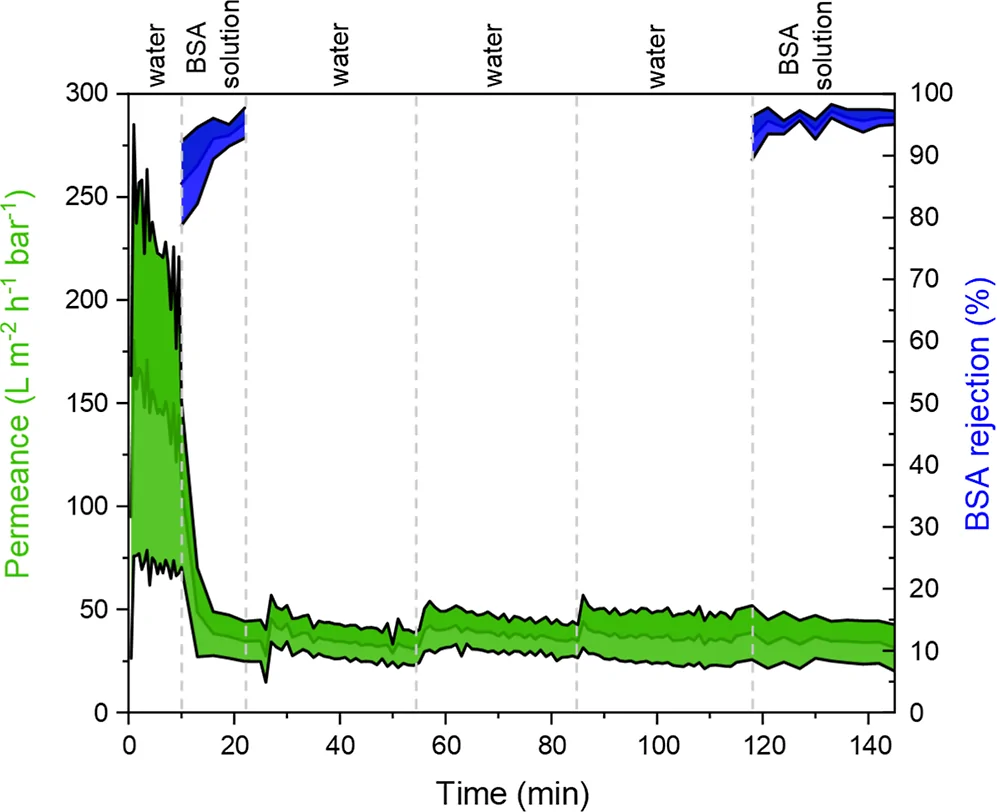
Cross-flow performance of the 15-0-T-25W75E membrane. The middle lines in the green and blue regions of the graph refer to the average permeance or BSA rejection, whereas the total green and blue regions refer to the error margins (±1 standard deviation). Vertical dashed lines indicate when the cell was depressurized, emptied, and refilled with a fresh solution.

### 3.4 Effect of Pore-Forming Agent Addition

Pore-forming agents (such as PEG) are frequently added to polymer dope solutions as they play a major role in enhancing pore formation, improving pore interconnectivity, and increasing hydrophilicity, and consequently improving the membrane permeability.^51–53^ Moreover, polar water-soluble pore-forming additives are reported to reduce the thermodynamic stability of polymeric dope solutions, making the binodal to be exceeded sooner.^55^ Here, to further explore improvement of performance of PSf membranes fabricated from TOU, the effect of a varying concentration of pore-forming agent PEG600 was evaluated (Figure 6, Table 5, SI 10). Low-molecular weight PEG was chosen due to good miscibility with TOU.

**Figure 6:**
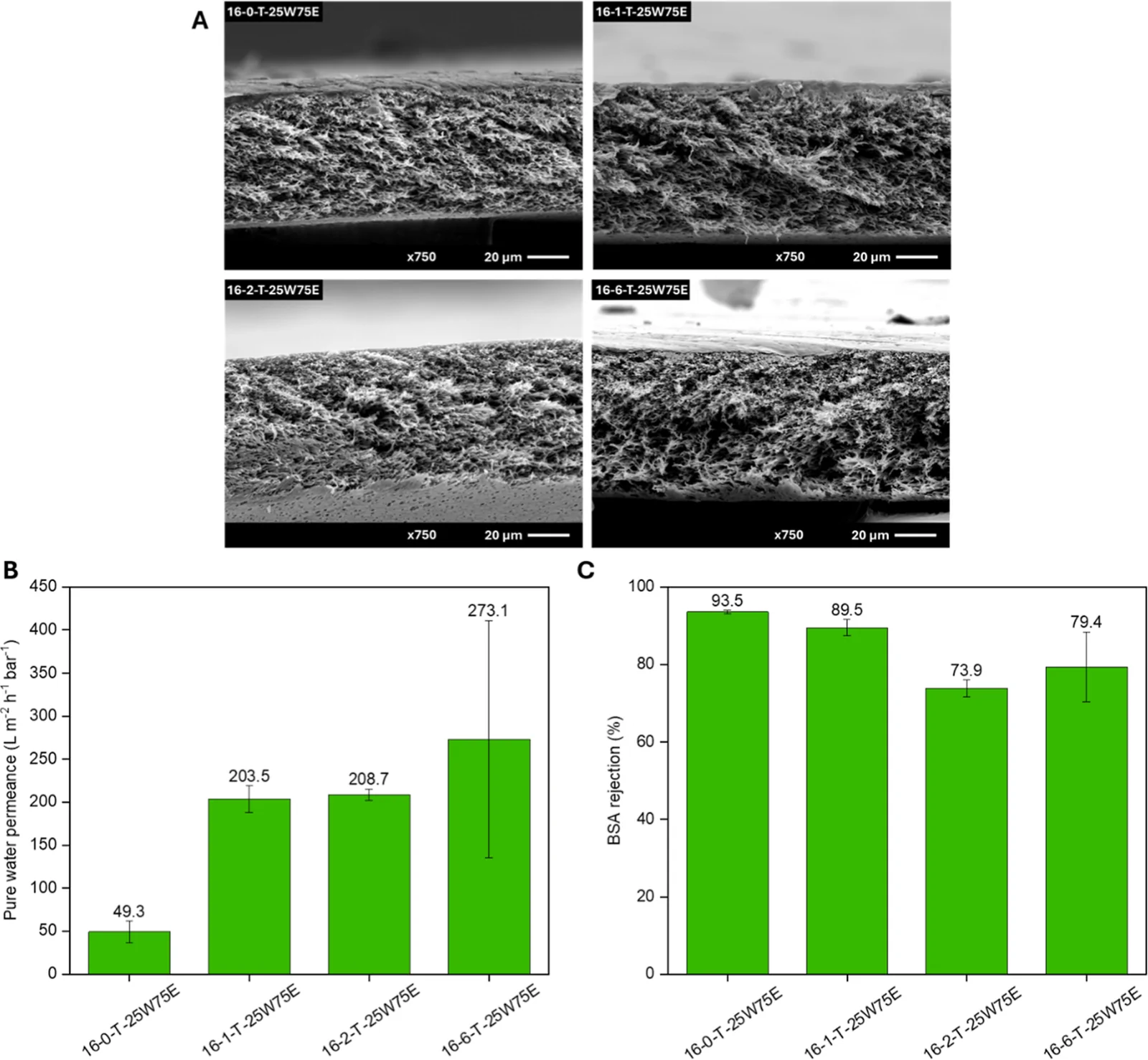
(A) Morphology of the PSf/TOU membranes with varying pore-forming agent concentrations (0, 1, 2, 6 wt %) in the dope solution. SEM images, full cross sections, magnification ×750. (B) Pure water permeance and (C) BSA rejection of PSf/TOU membranes with varying pore-forming agent concentrations in the dope solution.

**Table 5:**
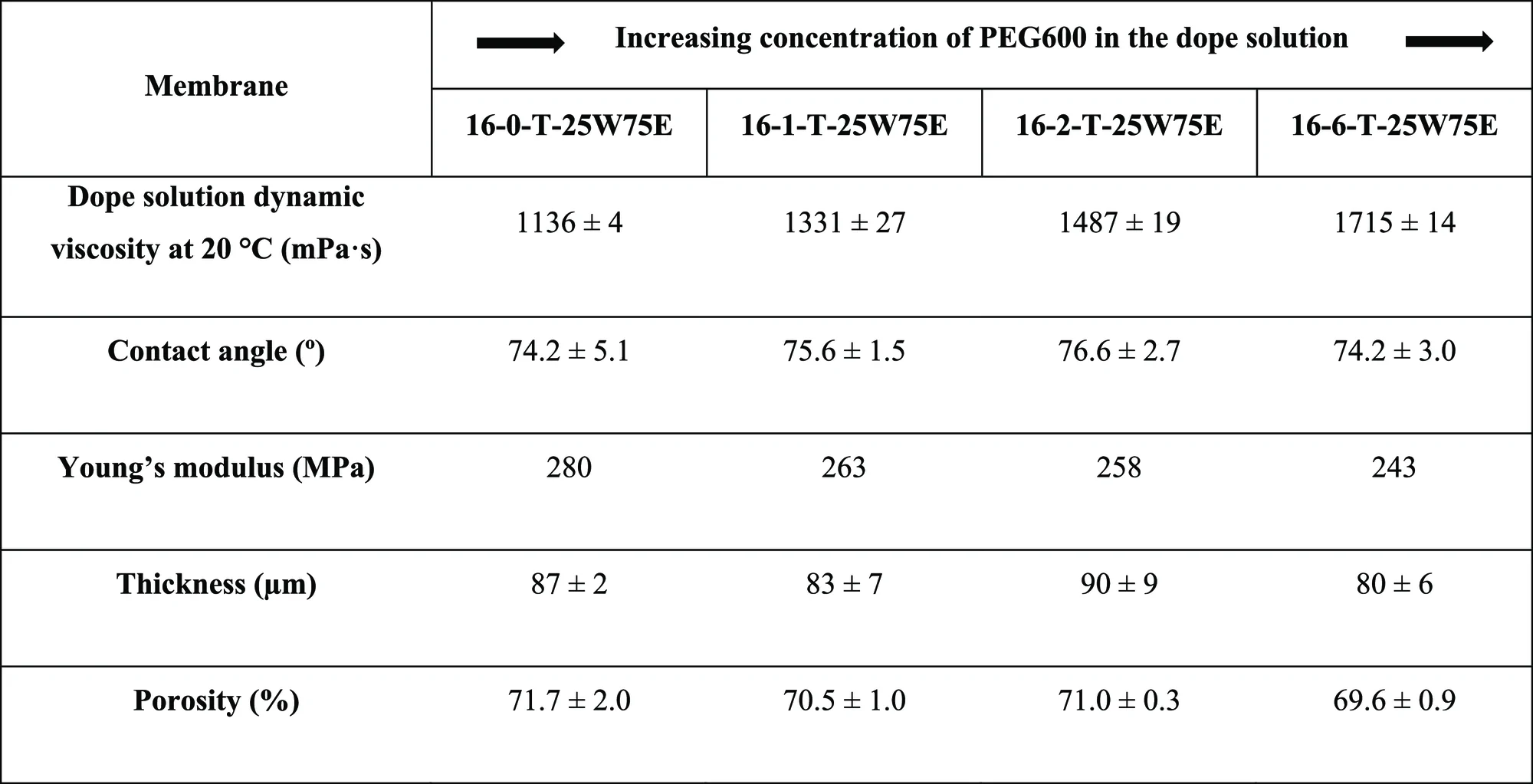
Properties (Viscosity, Contact Angle, Young’s Modulus, Thickness, Porosity) of PSf/TOU Dope Solutions and Membranes with Pore-Forming Agent Concentration in the Dope Solution

The widely reported trend of increasing permeability with PEG addition is also evident for PSf/TOU membranes, where addition of only 1 wt % PEG600 to the dope solution (16-1-T-25W75E) increases the pure water permeance (Figure 6B) more than 4-fold relative to the membrane without PEG600 additive (16-0-T-25W75E). BSA rejection of the membranes (Figure 6C) with PEG600 in the dope solution exhibits a decline in contrast with a membrane without any additives, 16-0-T-25W75E, showing that there is a trade-off between permeability and selectivity. Low-molecular weight PEG has been reported to act primarily as a pore former, enhancing water flux and reducing solute rejection.^55^ Generally, low-molecular weight PEGs such as PEG600 exhibit high mobility; hence, they can diffuse out easily during membrane solidification, leading to precipitation rate reduction and creation of a more permeable top layer.^56^ However, even considering the low-molecular weight, addition of PEG600 increases the dope solution viscosity, hence partially counteracting their pore-forming properties, which might explain why the resultant membrane pure water permeance does not linearly increase with a higher PEG600 content.

Based on Hansen solubility parameters and interaction distances, PEG600 is even more compatible with the 25:75 water/ethanol nonsolvent (*R*_*a*_ = 19.4 MPa^1/2^) than TOU (*R*_*a*_ = 15.1 MPa^1/2^), hence giving further evidence for more preferable leaching out to the coagulation bath, enhancing instantaneous demixing, and creating a membrane with higher top-layer porosity (i.e., higher permeability and slightly lower rejection). Moreover, PEG can act as a nonsolvent in the dope solution, which moves the dope solution closer to the binodal (Supporting Information 11), hence accelerating phase separation.^53^

Low-MW PEGs, like PEG600, have been reported not only as pore formers but also as macrovoid suppressors and agents increasing the hydrophilic character of membranes.^54^ However, no significant decrease in contact angle was observed (Table 5), hence PEG600 does not contribute to increase of hydrophilicity of the top layer. Moreover, in the case of PSf membranes formed from TOU dope solutions, structures both with and without the addition of porogen are macrovoid-free (Figure 6), similar to membranes in the Sections 3.1. and 3.3., hence PEG600 does not have a further contributing effect.

### 3.5 Comparison between PSf/TOU Membranes and Other Reported Lab-Scale PSf Membranes

In this work, a range of spongy macrovoid-free PSf membranes was fabricated using TOU as the solvent. The UF performance (pure water permeance vs BSA rejection) of the discussed membranes is collectively presented in Figure 7.

**Figure 7:**
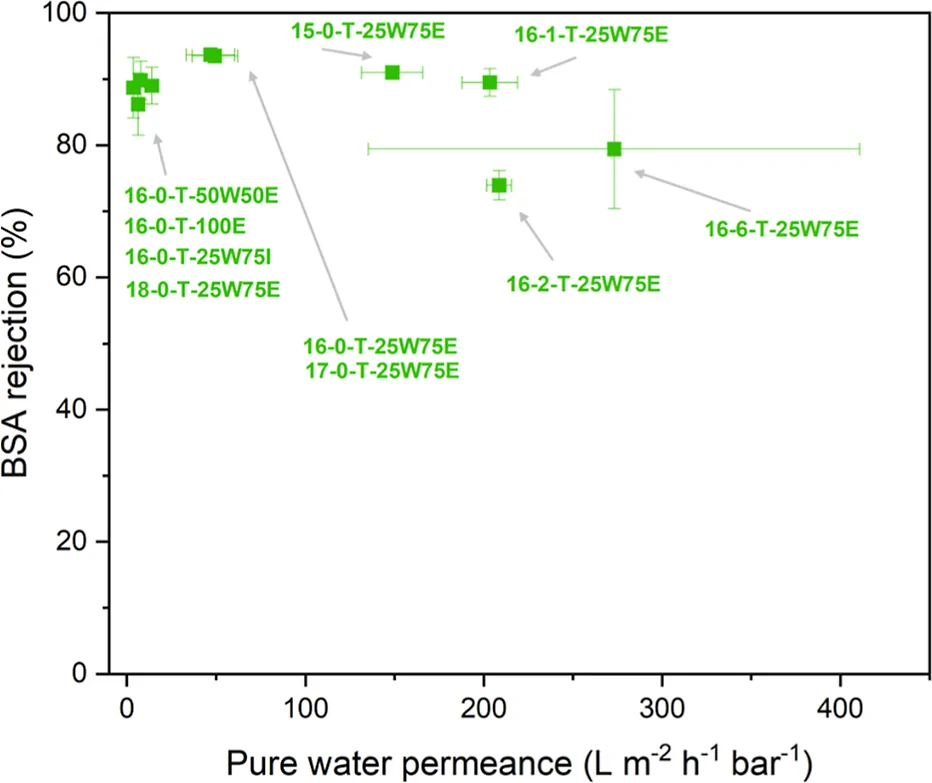
Comparison of performance of PSf membranes fabricated with TOU.

Typically, membranes have a selectivity-permeability trade-off.^57^ However, for highly efficient membrane separation processes, ideally, both high selectivity (in this case BSA rejection) and high permeability (in this case pure water permeance) are obtained. Therefore, out of the membranes from this work, in particular, the 15-0-T-25W75E and 16–1-T-25W75E membranes show superior performance, bearing both relatively high permeability and high selectivity, hence being promising UF membranes. It is worth noting that the presented filtration characterization was intended to be a proof-of-concept evaluation. While the results provide valuable insights into membrane performance, a more detailed characterization, including pore size distribution and the corresponding sieving curve, would further enhance our understanding of the separation mechanisms. However, such an investigation falls outside the scope of this research.

The PSf membranes fabricated from dope solutions containing TOU are also compared to other PSf UF membranes reported in the literature. Crucially, Table 6 provides a qualitative comparison of the orders of magnitude of permeances and rejections of other PSf NIPS membranes, rather than allowing for a direct distinction between membranes, as many differences in fabrication conditions are reported.

**Table 6:** Comparison of Best-Performing PSf Membranes Fabricated via NIPS with TOU as a Solvent (This Work) and Others Reported in Literature Using NMP and DMAc, Typically Used in Industrial Processes

PSf (wt·%)	additive in dope solution	solvent	nonsolvent	pure water permeability (L·m^–2^·h^–1^·bar^–1^)	BSA rejection (%)	reference
15	-	TOU	water/ethanol (25:75)	148.8 ± 17.1	91.0 ± 0.2	this work
16	1 wt·% PEG (MW 600)	TOU	water/ethanol (25:75)	203.5 ± 15.5	89.5 ± 2.1	this work
18		NMP	water	123.2	100	^58^
15	5 wt·% PEG, 6 wt·% UiO-66-(COOH)_2_	NMP	water	65.3	93.08	^59^
18	1 wt·% PVP, 0.025 wt·% i-GO	DMAc	water	135.0	95	^60^
22	22 wt·% PEG (MW 600)	DMAc	water	455.3	98.67	^61^
17.2	0.14 wt·% GO-PAMAM	NMP	water	124.5	99.53	^62^
18	0.1 wt·% TpPa nanofibers	DMAc	water	424.4	94.3	^63^
17.8	1 wt·% PVP (MW 10 000), 0.3 wt·% fullerol	NMP	water	191.9	93	^64^
18	4.8 wt·% PVP (MW 360 000), 2.1 wt·% IONPs-ALG	NMP	water	51.23	96.53	^65^
16	1 wt·% PVP, 2 wt·% PANI	DMAc	water	169	92	^66^

Membranes presented in this work are competitive with other membranes reported in the literature, hence showing promise as alternatives to currently produced membranes. It is worth noting that in the cited literature, PSf membranes are produced from dope solutions containing a variety of additives, including pore-forming agents (polyethylene glycol (PEG), polyvinylpyrrolidone (PVP)) and additives increasing hydrophilicity (graphene oxide, fullerol) to further enhance performance, whereas in this research also membranes prepared out of dope solution without additives show similar results. This work highlights the critical role of solvent–nonsolvent interactions in the NIPS process and successfully demonstrates, as a proof of concept, the potential of TOU in the fabrication of PSf membranes. Further optimization and the use of suitable additives could improve the membrane performance.

## 4 Conclusion

In this work, TOU, a polar aprotic ether-based solvent, was used for the first time for polymeric membrane fabrication via NIPS: production of PSf UF morphologies. The effect of processing parameters, i.e., composition of the coagulation bath (different water/ethanol mixtures), polymer concentration, and addition of pore-forming agent (PEG600) to the dope solution on membrane performance and properties (pure water permeance, BSA rejection, hydrophilicity, mechanical properties, thickness, porosity) was studied. The best-performing membranes, in terms of both permeance and rejection, were those prepared with either a lower PSf concentration or with added PEG600 and coagulated in a 25:75 water/ethanol mixture, specifically the 15-0-T-25W75E and 16-1-T-25W75E membranes.

Irrespective of processing parameters, PSf membranes fabricated with TOU exhibited a morphology with a denser selective top layer and a consistent spongy morphology of the substructure without the presence of macrovoids. This is in clear contrast to PSf membranes fabricated with NMP, which are characterized by substructures containing macrovoids. The suppression of macrovoids, when using TOU as a solvent, highlights the strong influence of the solvent choice on phase inversion behavior and on resultant membrane morphologies. The results suggest that both thermodynamic effects related to polymer–solvent–nonsolvent affinities, combined with kinetic effects concerning increased chain entanglement and dope solution viscosity, contribute to the formation of stable sponge-like morphologies during NIPS.

Further research should assess the practical scalability of TOU-based membrane fabrication, including potential for solvent recovery and recycling, membrane long-term operational stability, as well as techno-economic analysis (focusing on solvent price, commercial availability, and production scale).

Overall, our findings highlight the critical role of polymer–solvent–nonsolvent interactions in defining phase separation behavior and provide design guidelines for achieving stable spongy architectures in PSf membranes by introducing TOU to a portfolio of suitable solvents for NIPS. Introducing TOU expands the portfolio of suitable solvents for NIPS and offers a useful strategy for suppressing macrovoids in the PSf UF membranes.

## Supplementary Material



## Notes

Additional experimental details (raw data, SEM images, graphics, and setup photographs) and Hansen solubility parameters (PDF)
