# Supplementary material for: Designing Macrovoid-Free Polysulfone (PSf) Ultrafiltration (UF) Membranes with Tetraoxaundecane (TOU) Solvent
Source: ACS Appl Polym Mater. 2026 Sep 28;8(19):17530–44. doi: 10.1021/acsapm.6c02976 (PMC13660354; doi:10.1021/acsapm.6c02976)
Supplement: Supplementary file 1 [file ap6c02976_si_001.pdf]

Supporting information

Designing macrovoid-free polysulfone (PSf) ultrafiltration (UF) membranes with tetraoxaundecane (TOU) solvent

*Jadwiga Poniatowska, Kitty Nijmeijer, Zandrie Borneman**

* Corresponding author: z.borneman@tue.nl

Membrane Materials and Processes, Department of Chemical Engineering and Chemistry, Eindhoven University of Technology, PO Box 513, 5600 MB Eindhoven, The Netherlands

Supporting information 1

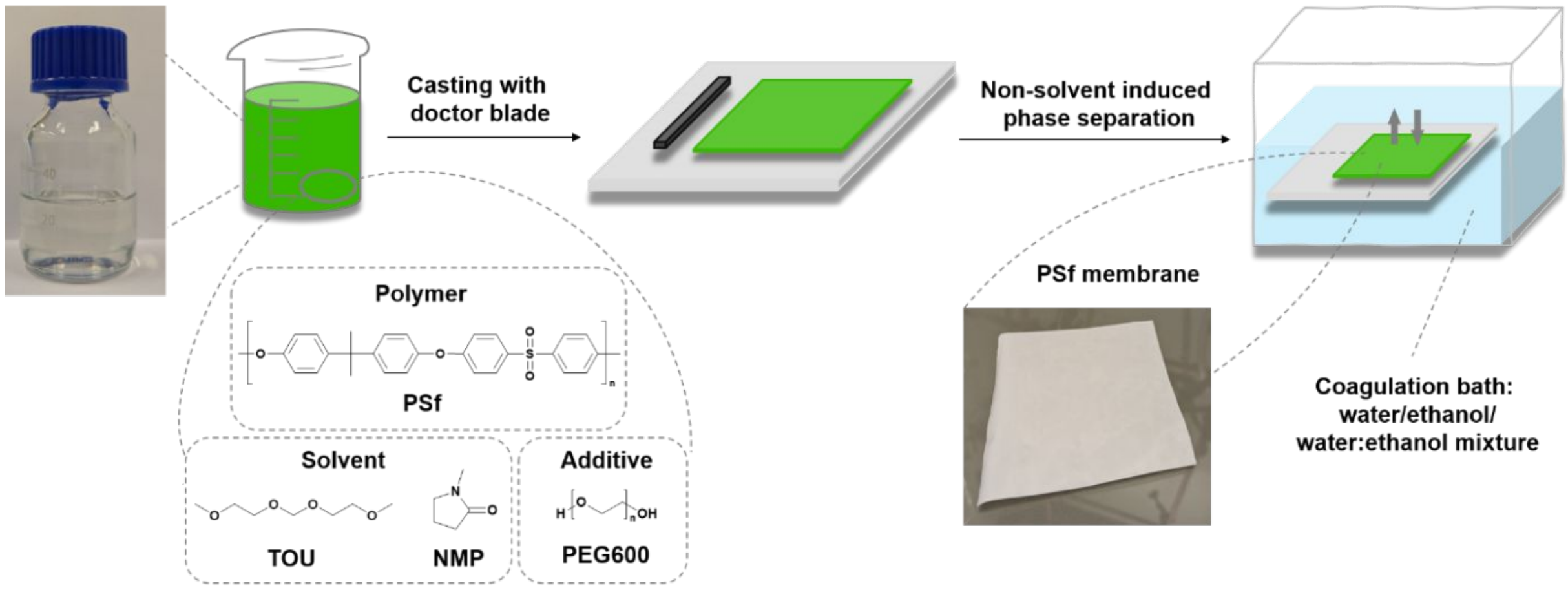

Figure 1: Schematic depiction of membrane fabrication process, including photos of the transparent PSf/TOU dope solution (top left) and the white homogenous PSf/TOU membrane (bottom right).

Supporting information 2

Table 1: Membrane codes and fabrication conditions.

Membrane abbreviation	Dope solution			Coagulation bath	
	PSf (wt%)	PEG (wt%)	Solvent	Water (v%)	Ethanol (v%)
**16-0-T-100W**	16	0	TOU	100	0
**16-0-T-75W25E**	16	0	TOU	75	25
**16-0-T-50W50E**	16	0	TOU	50	50
**16-0-T-25W75E**	16	0	TOU	25	75
**16-0-T-100E**	16	0	TOU	0	100
**15-0-T-25W75E**	15	0	TOU	25	75
**17-0-T-25W75E**	17	0	TOU	25	75
**18-0-T-25W75E**	18	0	TOU	25	75
**16-1-T-25W75E**	16	1	TOU	25	75
**16-2-T-25W75E**	16	2	TOU	25	75
**16-6-T-25W75E**	16	6	TOU	25	75
**16-0-N-100W**	16	0	NMP	100	0
**16-0-N-25W75E**	16	0	NMP	25	75

Supporting information 3

Table 2: Hansen solubility parameters for relevant compounds used for membrane fabrication.

	δ_D (MPa ½)	δ_P (MPa ½)	δ_H (MPa ½)	Reference
**PSf**	18.2	4.3	6.9	[1]
**TOU**	15.9	5.5	6.4	[2]
**NMP**	18.0	12.3	7.2	[3]
**PEG600**	16.6	3.2	12.1	[4]
**Water**	15.5	16.0	42.3	[5]
**Ethanol**	15.8	8.8	19.4	[5]
**IPA**	15.8	6.1	16.4	[5]

The Hansen solubility parameters of mixtures of compounds were calculated using Eq. 1:[6]

$$\delta_x = \Phi_1 \delta_{x1} + \Phi_2 \delta_{x2} \qquad \textit{Eq. 1}$$

where Φ_1 and Φ_2 are the volume ratios of the two components, δ_{x1} and δ_{x2} are the Hansen solubility parameters of the components (x refers to either D, P or H), and δ_x is the overall Hansen solubility parameter of the mixture.

Table 3: Hansen solubility parameters of relevant mixtures used for membrane fabrication.

	δ_D (MPa ½)	δ_P (MPa ½)	δ_H (MPa ½)
**75:25 water:ethanol**	15.6	14.2	36.6
**50:50 water:ethanol**	15.7	12.4	30.9
**25:75 water:ethanol**	15.7	10.6	25.1
**25:75 water:IPA**	15.7	8.6	22.9

Supporting information 4

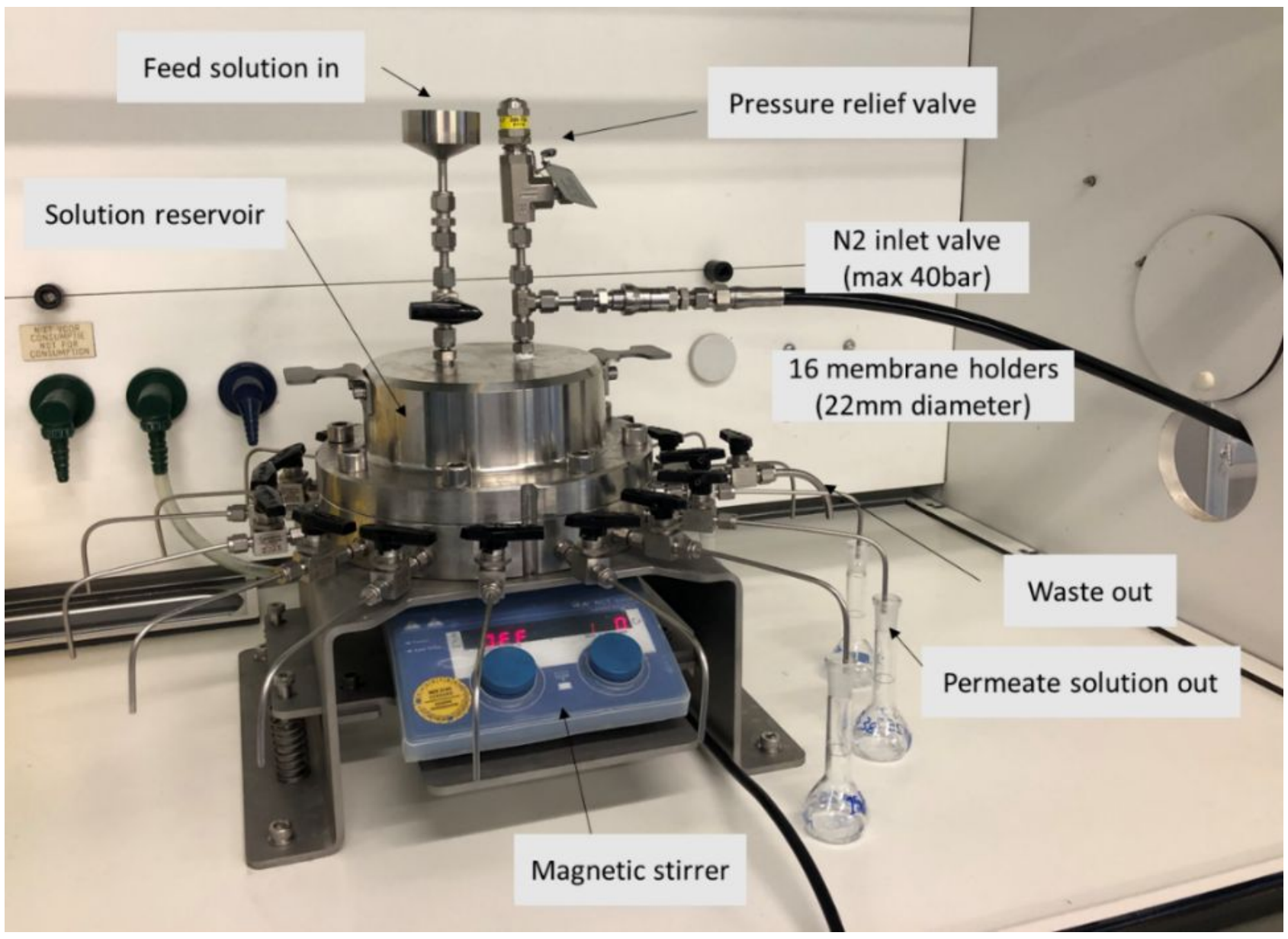

Figure 2: Photograph of a high-throughput dead-end filtration cell.

Supporting information 5

To assure that permeance was tested at steady-state conditions, a digital balance was used to monitor and record the mass of water permeated through 16-0-T-25W75E membrane in 1 minute intervals.

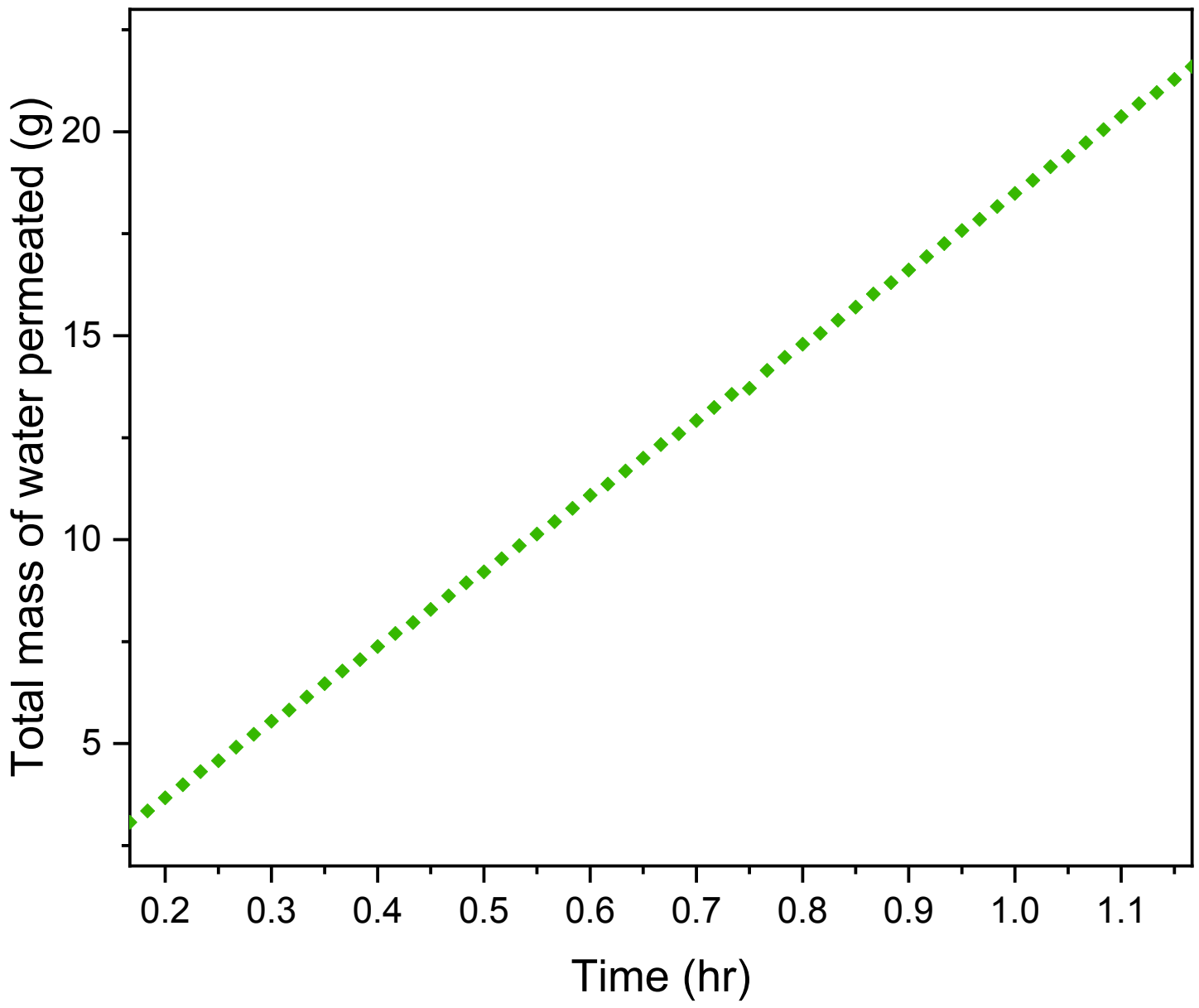

Figure 3: Graph of total mass of water permeated during the 1 h of measurement. A linear trend in the permeate mass shows that steady-state conditions are achieved.

Supporting information 6

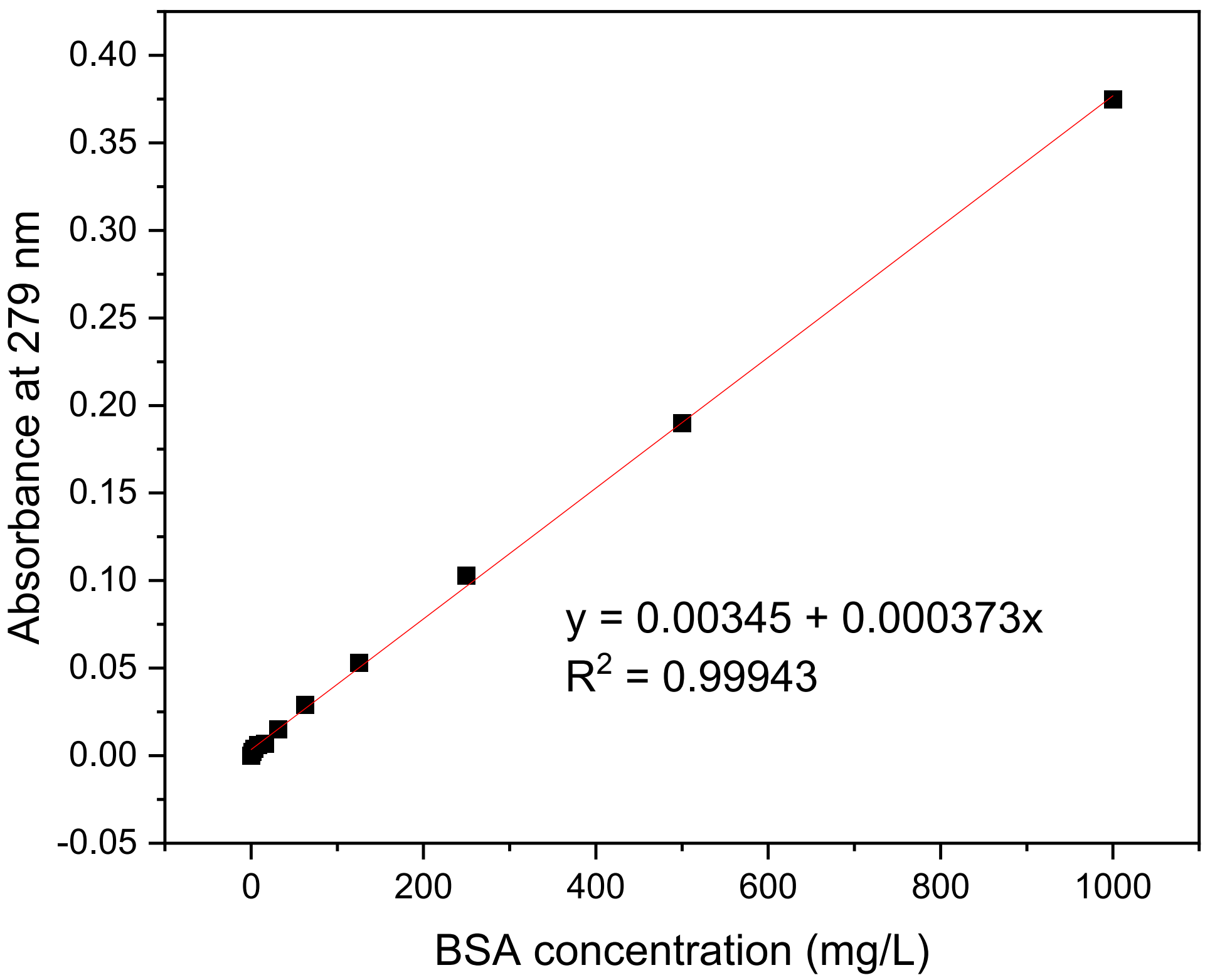

Figure 4: Calibration curve of BSA concentrations.

Supporting information 7

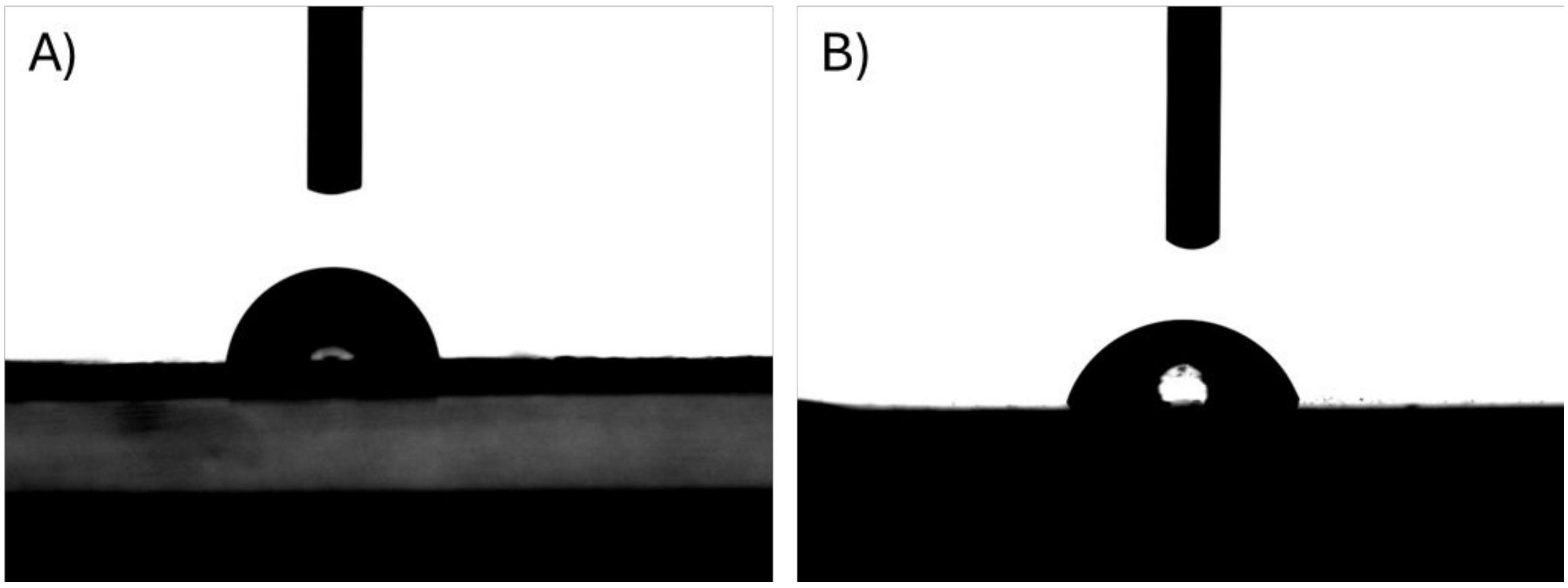

Figure 5: Examples of contact angle measurements of A) 16-0-T-100W membrane and B) 16-0-N-100W membrane.

Supporting information 8

The Young's modulus of the PSf membranes was calculated as the slope of the linear portion (between 0.4% and 1.6% strain) of the stress-strain curve.

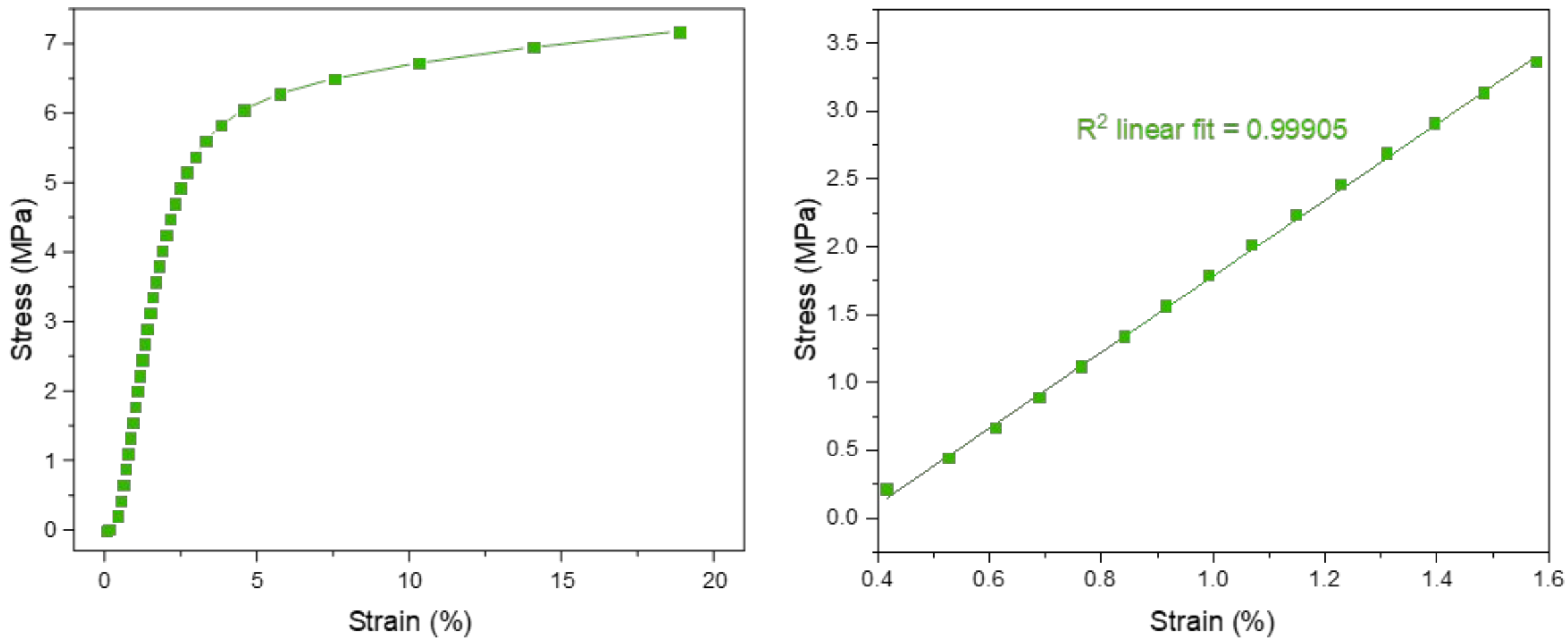

Figure 6: Example of a stress-strain curve of a 16-0-T-25W75E membrane. On the left: full curve, on the right: the linear portion of the graph including a line of best fit.

Supporting information 9

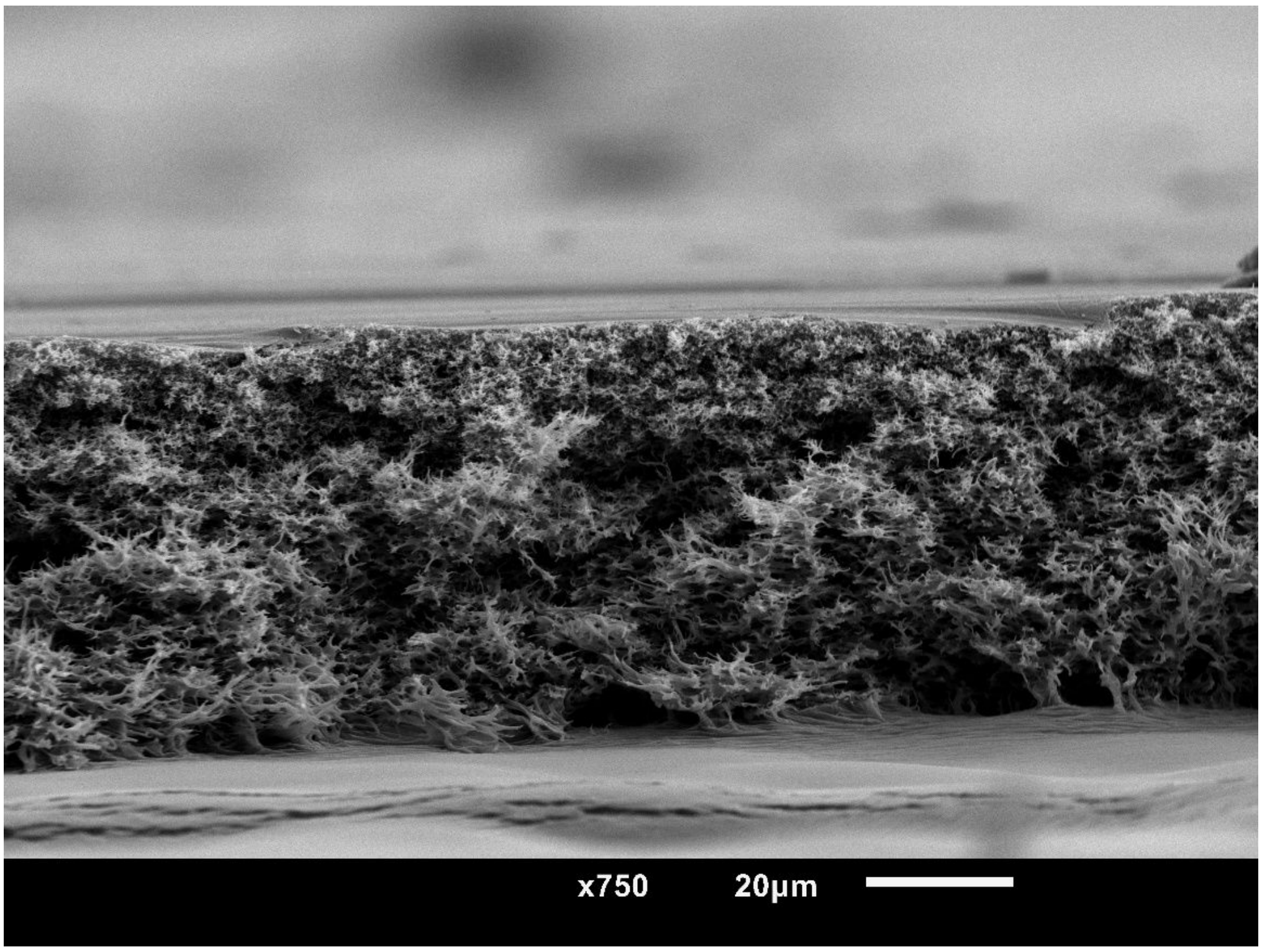

Figure 7: Morphology of the 14wt% PSf/TOU membrane coagulated in 25:75 water:ethanol bath. SEM image, full cross-section, magnification x750.

Supporting information 10

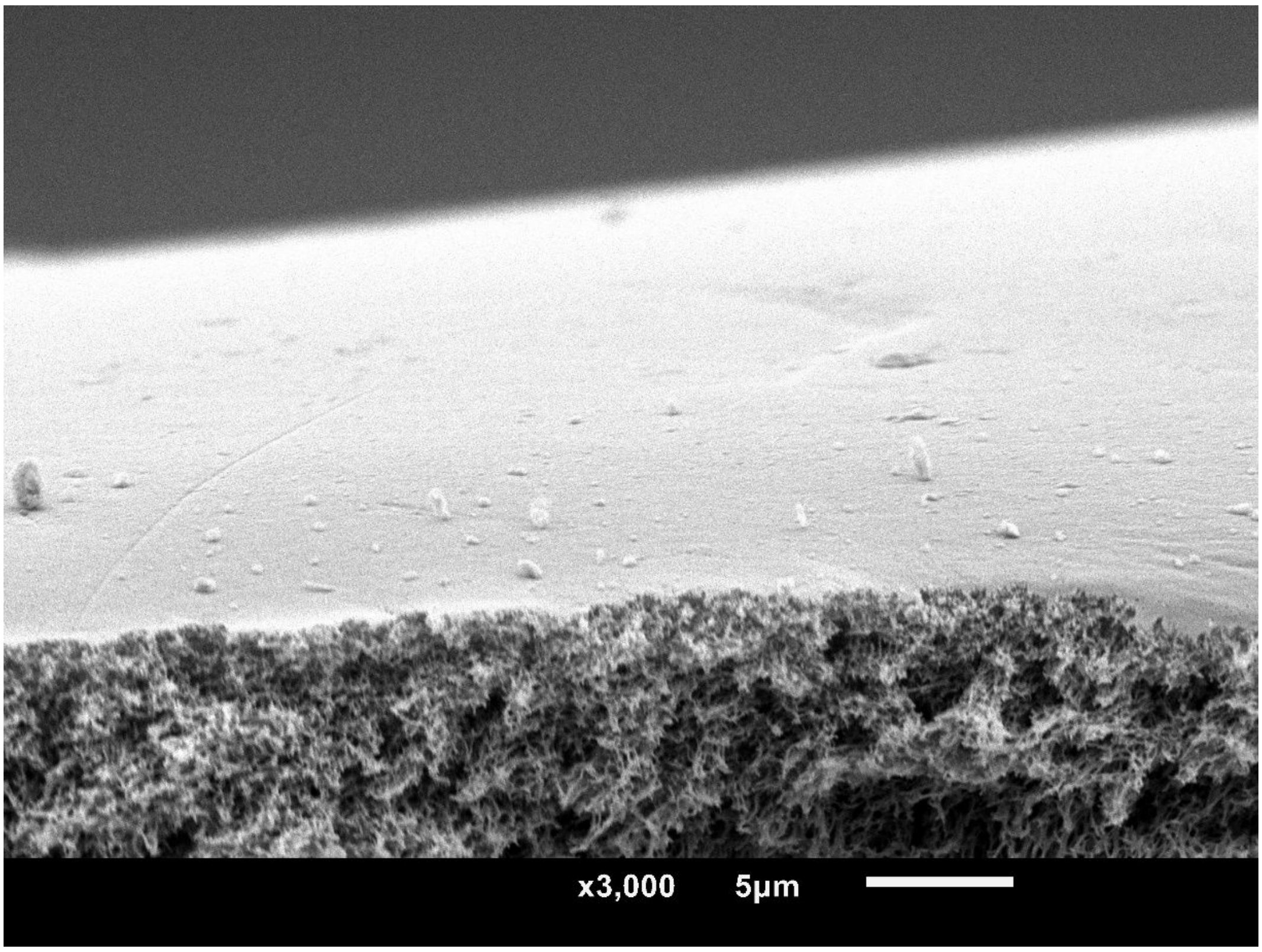

Figure 8: Morphology of the 16wt% PSf + 1wt% PEG600/TOU membrane coagulated in 25:75 water:ethanol bath. SEM image, cross-section of the top layer, magnification x3000.

Supporting information 11

Table 4: Raw data for cloud point measurements.

Compositions at which phase separation occurs

Water as non-solvent

Sample		g	wt%
4wt% PSf/TOU	PSf	1,6501	3,95
	Water	0,401	0,96
	TOU	39,684	95,09
	total	41,7351	
8wt% PSf/TOU	PSf	3,4483	7,89
	Water	0,5943	1,36
	TOU	39,684	90,75
	total	43,7266	
12wt% PSf/TOU	PSf	5,508	12,11
	Water	0,2995	0,66
	TOU	39,684	87,23
	total	45,4915	
16wt% PSf/TOU	PSf	7,5413	15,76
	Water	0,6111	1,28
	TOU	39,684	82,96
	total	47,8364	
20wt% PSf/TOU	PSf	9,9058	19,69
	Water	0,7172	1,43
	TOU	39,684	78,88
	total	50,307	

Ethanol as non-solvent

Sample		g	wt%
4wt% PSf/TOU	PSf	1,6481	3,94
	Ethanol	0,4969	1,19
	TOU	39,684	94,87
	total	41,829	
8wt% PSf/TOU	PSf	3,4568	7,87
	Ethanol	0,8028	1,83
	TOU	39,684	90,31
	total	43,9436	
12wt% PSf/TOU	PSf	5,4067	11,55
	Ethanol	1,7065	3,65
	TOU	39,684	84,80
	total	46,7972	
16wt% PSf/TOU	PSf	7,5595	15,54
	Ethanol	1,401	2,88
	TOU	39,684	81,58
	total	48,6445	
20wt% PSf/TOU	PSf	9,9304	19,27
	Ethanol	1,9052	3,70
	TOU	39,684	77,03
	total	51,5196	

Solutions with PEG

Sample		g	wt%
16wt% PSf/TOU	PSf	7,5413	15,76
	Water	0,6111	1,28
	TOU	39,684	82,96
	total	47,8364	
16wt% PSf + 1wt% PEG/TOU	PSf	7,6501	15,81
	Water	0,5801	1,20
	TOU	39,684	82,00
	PEG600	0,4788	0,99
	total	48,393	
16wt% PSf + 2wt% PEG/TOU	PSf	7,7874	15,86
	Water	0,641	1,31
	TOU	39,684	80,83
	PEG600	0,9825	2,00
	total	49,0949	
16wt% PSf + 6wt% PEG/TOU	PSf	8,1412	15,86
	Water	0,4419	0,86
	TOU	39,684	77,32
	PEG600	3,0547	5,95
	total	51,3218	

Solution with 25:75 water:ethanol

Sample		g	wt%
16wt% PSf/TOU	PSf	7,5415	15,77
	25:75 water:ethanol	1,1709	2,45
	TOU	39,684	82,96
	total	48,3964	

Bibliography

1 Avci, A. H., *et al.*, "Tuning the Electrochemical Properties of Novel Asymmetric Integral Sulfonated Polysulfone Cation Exchange Membranes," *Molecules 2021, Vol. 26, Page 265*, vol. 26, no. 2, p. 265, Jan. 2021, doi: 10.3390/MOLECULES26020265.

2 Lambiotte & Cie, "Acetals brochure ," 2019.

3 Dibrov, G., *et al.*, "Influence of Draw Ratio and Take-Up Velocity on Properties of Ultrafiltration Hollow Fiber Membranes from Polyethersulfone," *Fibers 2022, Vol. 10, Page 29*, vol. 10, no. 3, p. 29, Mar. 2022, doi: 10.3390/FIB10030029.

4 Liu, B., Du, Q., and Yang, Y. "The phase diagrams of mixtures of EVAL and PEG in relation to membrane formation," *J Memb Sci*, vol. 180, no. 1, pp. 81–92, Dec. 2000, doi: 10.1016/S0376-7388(00)00526-3.

5 Hansen, C. M. *Hansen Solubility Parameters*. CRC Press, 2007. doi: 10.1201/9781420006834.

6 Mihalovits, M. "Determination of the Hansen solubility parameters from solubility data using an improved evaluation approach, the concentric spheroids method," *J Mol Liq*, vol. 364, p. 119911, Oct. 2022, doi: 10.1016/J.MOLLIQ.2022.119911.
